# FAD/NADH Dependent Oxidoreductases: From Different Amino Acid Sequences to Similar Protein Shapes for Playing an Ancient Function

**DOI:** 10.3390/jcm8122117

**Published:** 2019-12-02

**Authors:** Lucia Trisolini, Nicola Gambacorta, Ruggiero Gorgoglione, Michele Montaruli, Luna Laera, Francesco Colella, Mariateresa Volpicella, Anna De Grassi, Ciro Leonardo Pierri

**Affiliations:** Laboratory of Biochemistry, Molecular and Structural Biology, Department of Biosciences, Biotechnologies, Biopharmaceutics, University of Bari, Via E. Orabona 4, 70125 Bari, Italy

**Keywords:** flavoprotein oxidoreductases, apoptosis-inducing factor (AIF), type II NADH dehydrogenase (NDH-2), thioredoxin reductase (TrxR1), dihydrolipoamide dehydrogenase (DLD), ubiquinone, molecular modeling, protein shape, antibiotics, mitochondrial respiration

## Abstract

Flavoprotein oxidoreductases are members of a large protein family of specialized dehydrogenases, which include type II NADH dehydrogenase, pyridine nucleotide-disulphide oxidoreductases, ferredoxin-NAD+ reductases, NADH oxidases, and NADH peroxidases, playing a crucial role in the metabolism of several prokaryotes and eukaryotes. Although several studies have been performed on single members or protein subgroups of flavoprotein oxidoreductases, a comprehensive analysis on structure–function relationships among the different members and subgroups of this great dehydrogenase family is still missing. Here, we present a structural comparative analysis showing that the investigated flavoprotein oxidoreductases have a highly similar overall structure, although the investigated dehydrogenases are quite different in functional annotations and global amino acid composition. The different functional annotation is ascribed to their participation in species-specific metabolic pathways based on the same biochemical reaction, i.e., the oxidation of specific cofactors, like NADH and FADH_2_. Notably, the performed comparative analysis sheds light on conserved sequence features that reflect very similar oxidation mechanisms, conserved among flavoprotein oxidoreductases belonging to phylogenetically distant species, as the bacterial type II NADH dehydrogenases and the mammalian apoptosis-inducing factor protein, until now retained as unique protein entities in *Bacteria/Fungi* or *Animals*, respectively. Furthermore, the presented computational analyses will allow consideration of FAD/NADH oxidoreductases as a possible target of new small molecules to be used as modulators of mitochondrial respiration for patients affected by rare diseases or cancer showing mitochondrial dysfunction, or antibiotics for treating bacterial/fungal/protista infections.

## 1. Introduction

### Flavoprotein Dehydrogenases

Several oxidative pathways depend on the ability of cells to oxidize NADH (reduced form of nicotinamide adenine dinucleotide cofactor), FADH_2_ (reduced form of flavin adenine dinucleotide cofactor), and ubiquinol (UQH_2_) by using dedicated enzymes known as FAD flavoproteins or flavoprotein oxidoreductases [1,2,3]. Flavoprotein oxidoreductases include pyridine nucleotide-disulphide oxidoreductases (glutathione reductases, trypanothione reductases, lipoamide dehydrogenases, mercuric reductases, thioredoxin reductases, alkyl hydroperoxide reductases), ferredoxin-NAD+ reductases (rubredoxin reductases, putidaredoxin reductases, terpredoxin reductases, and components of benzene 1,2-dioxygenases, toluene 1,2-dioxygenases, chlorobenzene dioxygenases, and biphenyl dioxygenases), NADH oxidases, and NADH peroxidases ( Inter PRO_ID: IPR023753) [4,5,6]. A widely studied subgroup within flavoprotein oxidoreductases consists of type II NADH dehydrogenases (known as NDH-2 in *Bacteria* and NDI in *Fungi*, [5]).

The great attention dedicated to type II NADH dehydrogenases is due to the fact that these proteins are widely present in bacteria, archaea, *Protista*, *Fungi*, and *Plants*, and appear to have no counterpart in *Animals* [7,8,9].

Thus, type II NADH dehydrogenases are considered crucial targets for antimicrobial therapies [10]. Conversely, it was recently shown that animal apoptosis-inducing factor (AIF) proteins are rotenone-sensitive NADH/ubiquinone oxidoreductases [11,12], raising the question about the opportunity to draw antibiotics against NDH-2 without considering a putative overlapping function with AIF.

All the cited flavoproteins are involved in the oxidation of NADH, through the reduction of FAD to FADH2 and its re-oxidation to FAD through the reduction of ubiquinone (UQ) to UQH2. Accordingly, both NDH-2- and AIF-crystallized structures show in their core a FAD molecule close to a NADH molecule. Notably, NDI from *Fungi* also shows a UQ molecule very close to the FAD molecule.

In some organisms, among the above cited species, complex I is missing (in some *Fungi*, i.e., in *Saccharomy cescerevisiae* [13], and, more in general, in *Bacteria*, as in *Staphylococcusaureus* [14]) and NDH-2 is the only active NADH dehydrogenase.

Impaired NADH oxidation in cells may determine a high NADH/NAD+ ratio, with a following increase in the production of reactive oxygen species (ROS), which may trigger apoptosis [15,16]. Thus, the regulation and maintenance of the proper NADH/NAD+ as well as the FADH_2_/FAD and UQH_2_/UQ ratios may be crucial for cell viability.

The presence of a FAD and a NADH molecule in both NDH-2/NDI and AIF proteins lets us suppose that AIF has a common functional ancestor with NDH-2 [6,17,18].

It was also recently proposed that the AIF bioenergetics function may be crucial for NADH oxidation alternative pathways [11,12], as well as for the mediation of caspase-independent apoptosis [19,20,21]. Indeed, AIF is anchored to the inner mitochondrial membrane protruding towards the mitochondrial intermembrane space of healthy cells [22]. After critical events governing the activation of various apoptotic pathways, allowing mitochondrial outer membrane permeabilization (MOMP), a protease (calpain or cathepsin) cleaves the AIF N-terminal domain (at residue number 102 [22]) and the cut C-terminal domain is released from the inner mitochondrial membrane, crosses the outer mitochondrial membrane, and translocates to the nucleus after association with macrophage migration inhibitory factor (MIF). In the nucleus, the AIF C-terminal domain associated with MIF mediates apoptosis participating in chromatin condensation and large-scale (∼50 kb) DNA degradation [19,23,24].

In this paper, we show that NDH-2/NDI from *Bacteria*/*Fungi* ( 5kmr.pdb from *Caldalkalibacillus thermarum* [10], 4g73.pdb from *S. cerevisiae* [25], and AIF from *Mammalia* ( 4bur.pdb from *Homo sapiens* [26]) share a very similar overall structure, able to accommodate FAD and NADH cofactors at similarly located binding regions. The shared cofactors and the corresponding binding regions indicate that the three enzymes should be able to drive the same oxidative reaction. Indeed, NDH-2 transfers an electron from NADH via FAD to UQ, without proton pumping [7,10]. At the same time, it is commonly accepted that NDI is able to transfer an electron from NADH via FAD to an UQ structurally related cofactor, behaving as a final electron acceptor [13]. Notably, along the crystallized multi-cofactor–NDI protein complex from *S. cerevisiae*, it is also possible to observe two UQ molecules in two close binding sites partially in contact with the FAD binding region [25]. Starting from the two observed poses, it was proposed that UQ_I_ (i.e., the UQ molecule closest to FAD) may interact with FAD that behaves as an intermediate molecule for electron transfer, and that NADH may transfer electrons via the FAD–UQ_I_ complex to UQ_II_

Similarly, AIF-crystallized structure hosts a FAD and two NADH molecules (4bur.pdb, [26]) and was recently proposed to be a rotenone-sensitive NADH: ubiquinone oxidoreductase [11,12]. Nevertheless, no semiquinone intermediate could be detected in the available AIF-crystallized structures, although such a neutral radical is expected to be produced after the transfer of a single electron. Our computational approach-based multistep analyses (according to [27]) for the comparison of sequences and crystallized structures of NDH-2/NDI and AIF proteins, has allowed the proposal of a putative binding region involved in direct interactions with UQ.

Furthermore, our comparative analysis has allowed an understanding that other FAD-dependent dehydrogenases, crucial for cell viability too, beyond AIF, NDI, and NDH-2, show a similar overall structure or protein shape [28,29].

Starting from this observation, it was possible to group the sampled/investigated structures in three main groups: AIF-like protein structures, NDH-2-like/NDI-like protein structures (each with specific features), and lipoamide dehydrogenase-like structures. All the members of the three main groups show a similarly located FAD-binding region, and for all of them, it is possible to propose a region putatively involved in NADH or UQ binding.

Last, but not least, our analyses allowed an exploration of putative binding regions located on AIF/NDI/NDH-2 like proteins, possibly involved in the binding of other protein subunits (i.e., cytochrome C, CytC), cofactors (i.e., Coenzyme A, CoA), and substrates (i.e., oxygen or H_2_S), as well as shedding light on possible species-specific FAD/NADH-dependent oxidoreductase-binding regions. Those binding regions may be targeted in the future with new high-affinity drugs, preventing side effects due to the simultaneous targeting of similarly located cofactor binding regions shared by bacterial/fungal/mammalia NDH-2/NDI/AIF-like proteins.

## 2. Materials and Methods

### 2.1. Protein SequenceSampling and Multiple Sequence Alignment (MSA)

AIF/NDH-2/NDI homologous sequences were collected from the RefSeq protein database using blastp searches (with default parameters). The sequence from *H. sapiens* AIF (NP_004199), *S. cerevisiae* NDI (NP_013586.1), and *C. thermarum* NDH-2 (WP_007502350.1) were used as queries to search for homologous sequences in selected species of animals, *Fungi*, *Plants*, and *Bacteria*. The selected taxonomic groups from *Bacteria* are *Bacillales* (TAX_ID 1385), *Enterobacteriales* (TAX_ID 91347), *Rhodospirillales* (TAX_ID 204441), *Rhodobacterales* (TAX_ID 204455), *Thermales* (gram-negative, TAX_ID 68933), and *Rhizobiales* (gram-negative, TAX_ID 356). Then, our searches were performed through other taxonomic groups, such as *Metazoa* (*Nematoda* (TAX_ID 6231), *Mammalia* (TAX_ID 40674), *Arthropoda* (TAX_ID 6656), *Anthozoa* (TAX_ID 6101)), *Fungi* (*Saccharomycetales*, TAX_ID 4892), and *Plants* (TAX_ID 3193), according to protocols described in [30]. The sampled sequences were retained whether they showed E-values lower than 10^-25^, query coverage higher than 70%, and the percentage of identical amino acids greater than 30%.

An MSA of the sampled sequences was built by using ClustalW implemented in the sequence editor package Jalview [31]. Redundant sequences with 100% identical amino acids were removed from the MSA.

### 2.2. Crystal Structure Sampling Via Folding Recognition

AIF/NDH-2/NDI homologus protein-crystallized structures were searched by using the folding recognition method implemented in pGenThreader [32]. The retrieved 49 crystal structures (those with “Certain” or “High” confidence level, according to [32] and [27]) were aligned, superimposed, and compared by using PyMOL [33].

Cofactor-binding regions were highlighted for comparative purposes by selecting residues within 4 Å from the cofactor crystallized in the sampled structures. When necessary, cofactors were also inserted in some crystal structures by comparative analyses and superimposition by using PyMOL according to what previously reported [27,30,34,35,36,37,38,39].

### 2.3. Phylogenetic Analysis

The analysis of the evolutionary relationships among the homologous sequences sampled by Blastp or among the sequences of the available homologous crystallized structures sampled by Folding Recognition was conducted using MEGA5 [40] starting from the MSA of the above cited 120 homologous protein sequences and from the MSA of the above cited 49 crystallized structure sequences.

In detail, the two trees were built from the ungapped MSA applying the maximum likelihood method with the JTT model for the amino acid substitutions and a gamma distribution (five discrete gamma categories) for the rates among sites. A total of 100 bootstrap samplings were applied to test the robustness of the tree (similar to what was described in [30]).

## 3. Results

### 3.1. Evolutionary Relationships among the Sampled AIF/NDH-2/NDI Homologous Sequences

Although it is commonly accepted that all NDH-2/NDI dehydrogenase sequences might share a common ancestral sequence, based on their conserved similar function and overall structure [13], it has recently been proposed that AIF has a function similar to NDH-2/NDI, i.e., that it is a rotenone-sensitive NADH:UQ oxidoreductase [11,12].

**Figure 1:**
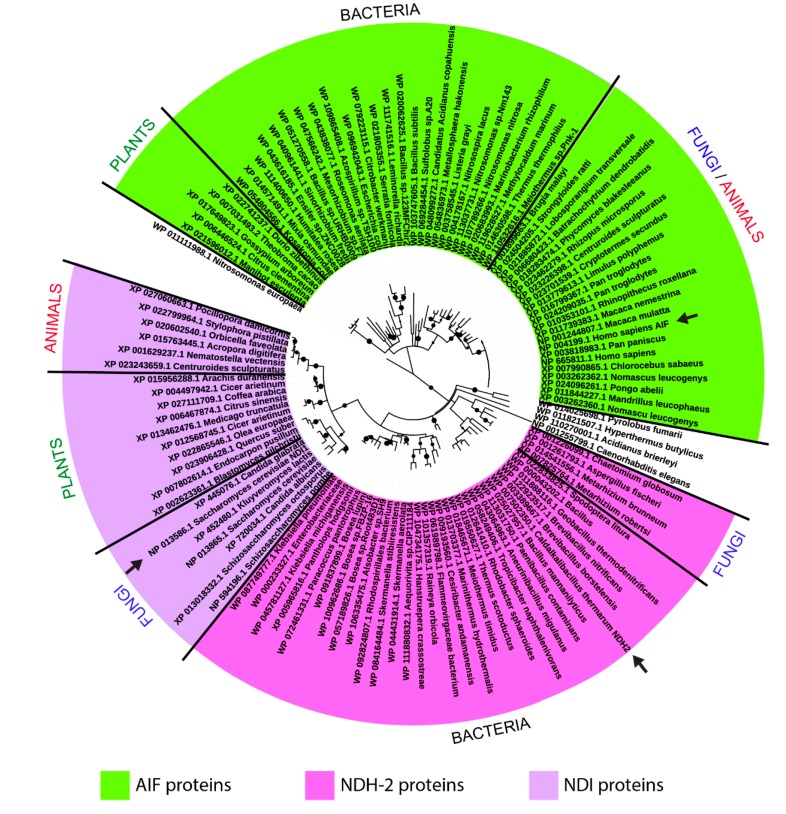
Phylogenetic tree of AIF/NDI/NDH-2-homologous sequences. Maximum likelihood phylogenetic tree of AIF/NDH2/NDI-homologous protein sequences selected from representative species of bacteria, *Fungi*, *Plants*, and animals. Each one of the tree leaves reports the corresponding organism and RefSeq protein accession number. Arrows indicate the query-protein sequences used for sampling all the other homologous sequences. Nodes supported by bootstrap values greater than 0.7 are indicated as black dots.

However, the spread of these oxidoreductases in distant taxonomic groups of species and their evolutionary relationships are currently unknown. Thus, we searched for proteins sharing sequence similarity with AIF or NDH-2 or NDI in representative species selected from bacteria, *Plants*, *Fungi*, and animals, and we found 120 putative homologous proteins. The evolutionary relationships among all these proteins showed two main clusters referring to AIF-homologous proteins and NDH-2/NDI-homologous proteins, whereas the latter were further divided into two main subclusters (Figure 1). AIF-homologous proteins were detected in members from all four taxonomic groups while NDH-2-homologous proteins were detected in bacterial and fungal species, and NDI-homologous proteins were detected in fungal, plant, and animal species (Figure 1).

In detail, close to the *Mammalia* AIF sequences, it is possible to observe a group of *Nitrosomonadales* and *Thermales* sequences that group into a subcluster adjacent to the AIF subcluster *Metazoan* sequences. Then, in the same tree-region, a bit more distant but related to the AIF-like sequence cluster, it is possible to observe a sequence cluster hosting *Bacillales*, *Sulfolobales*, and *Plant* sequences (i.e., XP_022741237.1_*Durio_zibethinus*, Figure 1).

On the other side of the tree, it is possible to observe a cluster of *Bacillales* NDH-2-like sequences clustering together with NDH-2-like sequences from *Fungi* together with two subgroups of *Bacteria* sequences (the first consisting of *Enterobacteriales*, *Rhodospirillales*, *Rhodobacterales*, and *Rhizobiales*, and the second consisting of *Thermales*, *Flavobacteriales*, and *Cytophagales* sequences) related to NDH-2-like *Bacillales* sequences. Finally, a last subgroup containing *Fungi*, *Plant*, and *Anthozoa* NDI-like sequences is detectable, in the same tree region but separated from the NDH-2-like sequence cluster (Figure 1).

### 3.2. Sequence Features of the Sampled AIF/NDH-2/NDI Protein Sequences

It was expected that the proposed specialization in species-specific biochemical pathways should reflect the existence of further well-conserved sequence features observable by building a MSA, despite the global highly variable percentage of identical residues (in the 15% to 99% range) shared by the sampled full-length sequences (Supplementary Table S1).

**Figure 2:**
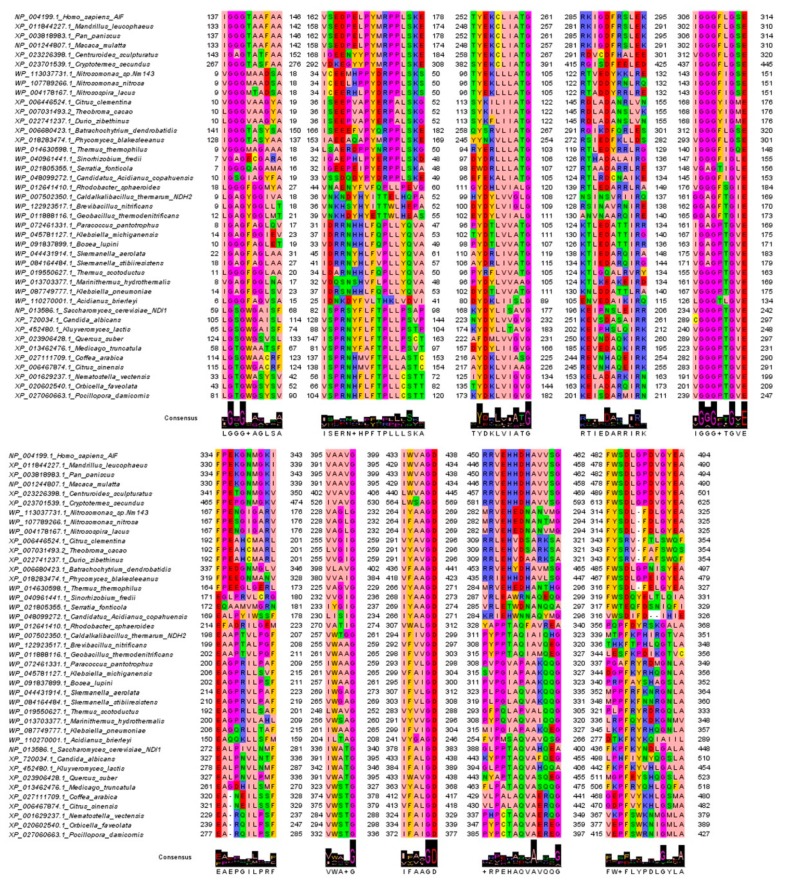
AIF/NDI/NDH-2-conserved sequence motifs. Sequence motifs detected by comparing the sampled AIF/NDI/NDH-2 homologous sequences sampled by blastp. The highlighted motifs reveal crucial protein regions involved in cofactor binding and/or the protein function mechanism. The jalviewzappo color style was used for coloring amino acids. Logo representation is also reported to highlight the most conserved residues.

In fact, by inspecting the MSA of AIF/NDI/NDH-2-sampled homologs, it is possible to recognize 10 amino acid conserved motifs. Some of those motifs, i.e., the two glycine-rich motifs (respectively the first and fifth sequence motif grouped in Figure 2) and the eighth motif, are conserved in all the investigated sequences, underlining a putative common involvement in the protein function/mechanism. The other sequence motifs show a high degree of amino acid conservation within specific taxonomic groups, reflecting a species-specific acquired function or substrate/cofactor specificity/affinity (Figure 2).

In detail, the most conserved protein regions, at the first and the fifth motifs, consist of two glycine-rich regions strongly conserved in all the sampled sequences. As an example, in metazoa, the two glycine-rich motifs are at the level of residues 137-IGGGTAAFA-146 and 306-IGGGFLGSE-314 (*H. sapiens* numbering) and align with residues 9-LGAGYGGIV-17 and 161-IGGAGFTGE-169 in *Bacteria* (*C. thermarum* numbering) and 59-LGSGWGAIS-67 and 234-VGGGPYGVE-242 in *Fungi* (*S. cerevisiae* numbering) (Figure 2). It is interesting to note that the first highlighted motif hosts an aromatic residue in half of the sampled sequences, whereas the sixth motif always hosts an acidic residue and a further aromatic residue in most of the sampled sequences.

The second highlighted sequence motif, located at residues 162-VSEDPELPYMRPPLSKE-178 (*H. sapiens* numbering), close to the first glycine-rich motif, appears to be conserved in specific taxonomic groups within the sampled *Mammalia* AIF sequences. This motif appears to be conserved, with few differences in the AIF-like sequences sampled through *Arthropoda*, *Fish*, *Fungi*, *Plants*, and AIF-closest sampled *Bacteria* sequences (Figure 2). The motif 162-VSEDPELPYMRPPLSKE-178 (*H. sapiens* numbering) of AIF-like sequences aligns with the motif 37-NKNDYHYITTELHQPAA-53 (*C. thermarum* numbering) observed in NDH-2 from *Bacillales* and other *Bacteria* (i.e., 31-IDRRNHHLFQPLLYQVAT-48, *Paracoccus pantotrophus* numbering), such as in *Rhodobacterales*, *Rhizobiales*, *Enterobacteriales*, *Thermales*, and *Rhodospirillales*. These motifs align with the NDI highly conserved motif 83-SPRSYFLFTPLLPS-96 (*S. cerevisiae* numbering) observed in *Fungi* and NDI-like sequences sampled from *Plants*, i.e., 82-SPRNYFAFTPLLPS-95 (*Medicago truncatula* numbering).

Two further highly conserved motifs, i.e., the third and fourth, consist of residues 252-TYEKCLIATG-261 (*H. sapiens* numbering), with the last glycine conserved in all the sampled sequences, and 285-RKIGDFRS-292 (*H. sapiens* numbering), respectively. The third motif 252-TYEKCLIATG-261 (*H. sapiens* numbering) is conserved in all the sampled *Mammalia* AIF sequences. The same motif with few variations is observed in *Arthropoda* 258-TYEKCLIATG-267, AIF-like sequences from *Bacteria* 96-TYEKLLLATG-105 (*Nitrosomonas nitrosa* numbering), *Plants* 113-SYKILIIATG-122 (*Citrus clementina* numbering), *Fungi* 258-QYSRVLIATG-267 (*Batrachochytrium dendrobatidis* numbering), and several groups of AIF-like proteins from *Bacteria*, i.e., *Nitrosomonadales* 96-TYEKLLLATG-105 (*N. nitrosa* numbering), and NDH-2-like sequences from *Rhodospirillales* 100-SYDRLVLATG-109 (*Roseomonasaerilata* numbering), *Thermales* 94-RYERLLLATG-103 (*Thermus thermophilus* numbering), *Rhodobacterales* 111-GYDHLVIALG-120, *Rhizobiales* 97-DYDRLLLATG-106 (*Sinorhizobiumfredii* numbering), *Enterobacteriales* 98-EWDRLLLATG-107 (*Serratia fonticola* numbering), *Sulfolobales* 96-EFEKALIATG-105 (*Candidatusacidianus* numbering), and *Bacillales* 99-HYDYLVVGLG-108 (*C. thermarum* numbering).

A very similar motif is also observed in NDI-like sequences from *Fungi* 214-KYDYLVVGVG-223 (*S. cerevisiae* numbering)*, Plants* 216-AYDKLVIASG-225 (*Coffeaarabica* numbering), and *Anthozoa* 125-IYDKLVIGVG-134 (*Nematostella vectensis* numbering).

The fourth motif 285-RKIGDFRS-292 (*H. sapiens* numbering) aligns with a different conserved motif in NDH-2 sequences from *Rhodobacterales* 124-KTLEDATT-131 (*P. pantotrophus* numbering), *Enterobacterales* 130-KTLEDATT-137 (*Klebsiella michiganensis* numbering), and *Rhizobiales* 126-KTLEDATT-133 (*Bosealupini* numbering), with few variations in *Rhodospirillales* 138-KTIEDARQ-145 (*Skermanella aerolata* numbering), *Thermales*, and *Bacillales* 128-RSINSVRL-135 (*Pseudochelatococcus contaminans* numbering) or 127-NSINSVRI-134 (*C. thermarum* numbering).

This motif is also observed with few variations in NDI from *Fungi* 196-KEIPNSLEI-204 (*S. cerevisiae* numbering), NDI-like sequences from *Plants* 185-KEVEDAQK-192 (*M. truncatula* numbering), and *Anthozoa* 153-KELADARK-160 (*N. vectensis* numbering).

The five last conserved motifs are detectable in all the sampled sequences.

A sixth motif conserved with specific variations in at least two great taxonomic groups consists of amino acids 334-FPEKGNMGKI-343 (AIF, *H. sapiens* numbering) highly conserved in AIF from *Mammalia* and *Arthropoda* (341-FPETGNMGKV-350, *Centruroides sculpturatus* numbering) and AIF-like proteins from some *Plants* (192-FPEAHCMARL-201, *C. clementina* numbering), *Fungi* (319-FPEEGNMANV-328, *Phycomyces blakeseeanus* numbering), and *Bacteria* (167-FPESGIGARV-176, *N. nitrosa* numbering).

The sixth motif 334-FPEKGNMGKI-343 (AIF, *H. sapiens numbering*) aligns with a highly conserved motif observed in *Fungi* NDI, i.e., 272-EALPIVLNMF-281 (*S. cerevisiae* numbering), and in NDI-like proteins from *Plants* (327-EALPNVLPMF-336, *Quercus suber* numbering) and *Anthozoa* (229-EA-RQILPSF-237, *N. vectensis* numbering).

The above cited motifs align with a highly conserved motif detectable in NDH-2 from *Bacillales* 198-EAAPTVLPGF-207 (*C. thermarum* numbering), from *Rhodobacterales* 200-EAGPRILPAF-209 (*P. pantotrophus* numbering), from *Enterobacteriales* 206-EAGPRLLSVF-215 (*K. michiganensis* numbering), from *Rhodospirillales* 214-EAGPRVLPAF-223 (*S. aerolata* numbering), from *Rhizobiales* 202-EAGPRILPSF-211 (*B. lupini* numbering), and *Thermales* 192-EAGPRLLSAF-201 (*Thermus scotoductus* numbering)

The seventh motif consists of residues 395-VAAVG-399 (*H. sapiens* numbering) aligning with, NDH-2 from *Bacteria*, i.e., 257-VWTGG-299 (*C. thermarum* numbering) or NDI from *Fungi* 336-IWATG-340 (*S. cerevisiae* numbering). Notably, the final G is conserved in all the sampled sequences.

The eighth motif consists of residues 433-IWVAGD-438 (*H. sapiens* numbering) aligning with NDH-2 from *Bacteria*, i.e., 294-IFIVGD-299 (*C. thermarum* numbering) or NDI from *Fungi* 378-IFAIGD-383 (*S. cerevisiae* numbering). Notably, the final GD dipeptide is conserved in all the sampled sequences.

The ninth motif consists of residues 450-RRVEHHDHAVAVSG-459 (*H. sapiens* numbering) that aligns with a different motif conserved with few variations in NDI-sequences from *Fungi* 388-GLPPTAQVAHQEA-400 (*S. cerevisiae* numbering) and NDH-2 sequences from *Bacteria* 311-PYPPTAQIAIQHG-323 (*C. thermarum* numbering).

The tenth motif consists of residues 482-FWSDLGPDVGYEA-494 (*H. sapiens* numbering) that aligns with a similar motif conserved with few variations in NDI-sequences from *Fungi* 436-FKPFKYNDLGALA-448 (*S. cerevisiae* numbering) and NDH-2 sequences from *Bacteria* 339-MTPFKPHIRGTVA-351 (*C. thermarum* numbering). Notably the last alanine residue is conserved in all the sampled sequences.

### 3.3. Comparative Analysis of AIF, NDH-II, NDI, and the pGenTHREADER-Suggested Template Proteinsfor Comparative Modeling

#### 3.3.1. Superimposition of AIF, NDI, and NDH-2

**Figure 3:**
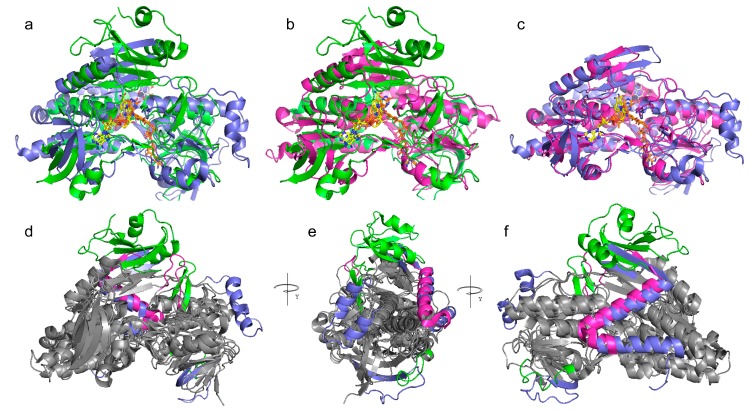
Comparative analysis of AIF/NDI/NDH-2 3D structure. Panels (**a**,**b**,**c**) Lateral view of superposition of *H. sapiens* AIF (in green cartoon, 4bur.pdb) and *S. cerevisae* NDI (in blue cartoon, 4g73.pdb) that gives a root mean square deviation(RMSD) of 2.891 Å; *H. sapiens* AIF (4bur.pdb) and *C. thermarum* NDH2 (in magenta cartoon, 5kmr.pdb) that gives an RMSD of 1.891 Å; *S. cerevisae* NDI (4g73.pdb) and *C. thermarum* NDH2 (5kmr.pdb) that gives an RMSD of 1.384 Å. Panel (**d**,**e**,**f**) Three 90-degree rotation of the alignment view of 4bur, 4g74, and 5kmr. In the grey cartoon representation, the common features highlighted by the superposition of the three structures. Specific AIF/NDI/NDH-2 structural features highlighted by the proposed superposition are reported in green, blue, and magenta cartoon representations. All the three aligned proteins are represented in complex with the FAD (orange sticks, panels **a**–**c**), NAD^+^ (yellow sticks, panels **a**–**c**), and UQ (white sticks, panels **a** and **c**) cofactors, when the cited cofactors were present in the reported crystallized structures.

Given the existence of the crystallized structures of the human AIF (4bur.pdb), *S. cerevisiae* NDI (4g73.pdb), and the *C. thermarum* NDH-2 (5kmr.pdb), we compared their structures by superimposition and we observed that beyond the shared motifs the investigated proteins show a highly similar overall protein structure and shape (Figure 3).

The main differences between the three crystallized structures are in the C-terminal domain and in some loops. Indeed, the AIF structure has a longer C-terminal domain containing an extra helix domain (residues 497–612, *H. sapiens* 4bur.pdb numbering) at variance with the shorter C-terminal domains of NDI (residues 458–513, *S. cerevisiae* 4g73.pdb numbering) and NDH-2 (residues 363–396, *C. thermarum* 5kmr.pdb numbering).

Furthermore, AIF, NDI, and NDH-2 show one or two specific loops. AIF-specific loops are observed at the level of residues 210–222 and 440–451 (4bur.pdb numbering, Figure 3), whereas two specific loops are observed in the NDI structure at the level of residues 138–163 and 413–435 (4g73.pdb numbering, Figure 3). A specific loop is observed at the level of residues 302–312 of NDH-2 (5kmr.pdb numbering, Figure 3).

#### 3.3.2. Sampling of Homologous-Crystallized Structures by Folding Recognition Methods

Along the Blastp sequence sampling, it was also noticed that several dehydrogenases out of the sampled homologous protein sequences were annotated as dehydrogenases with different specific functions. i.e., among the sampled homologous AIF/NDH-2/NDI proteins, it was possible to detect a FAD-dependent pyridine-nucleotide disulfide oxidoreductase [41] from *N. nitrosa* (WP_107789266.1) and a ferredoxine reductase [42] from *Nitroso spiralacus* (WP_004178167.1), grouping in a subcluster adjacent to AIF sequences sampled from Mammalia. Another example is observed in a cluster adjacent to AIF-like sequences, where it is possible to observe a monodehydroascorbate reductase [43] from *D. zibethinus* (XP_022741237.1) proposed to be located within peroxisomes.

To verify if some structures of the above cited proteins were available in the protein data bank and showed an overall structure similar to AIF/NDH-2/NDI protein structures, we searched for AIF/NDH-2/NDI 3D-homologous structures by using the fold recognition tools implemented in pGenTHREADER.

In this way, several FAD/NAD(P)H-dependent dehydrogenase-crystallized structures, proposed to have a structure homologous to that observed for AIF, NDI, and NDH-2, were sampled. The sampled structures from different species were available in the protein data bank under different functional annotations (Table 1).

Despite the low percentage of identical amino acids showed by sequences sampled by pGenTHREADER with AIF (4bur), NDI (4g73), and NDH-2 (5kmr) sequences (ranging between 15% and 30%, Supplementary Table S2), the sampled crystallized structures showed an overall structure highly similar to that observed for AIF-, NDI-, and NDH-2-crystallized structures. The structural similarity among the sampled structures was quantified by visual inspection and by estimating the RMSD of AIF/NDI/NDH-2 3D coordinates and coordinates of the sampled structures (ranging between 0.8 and 4.5 Å, Table 1). 

**Figure 4:**
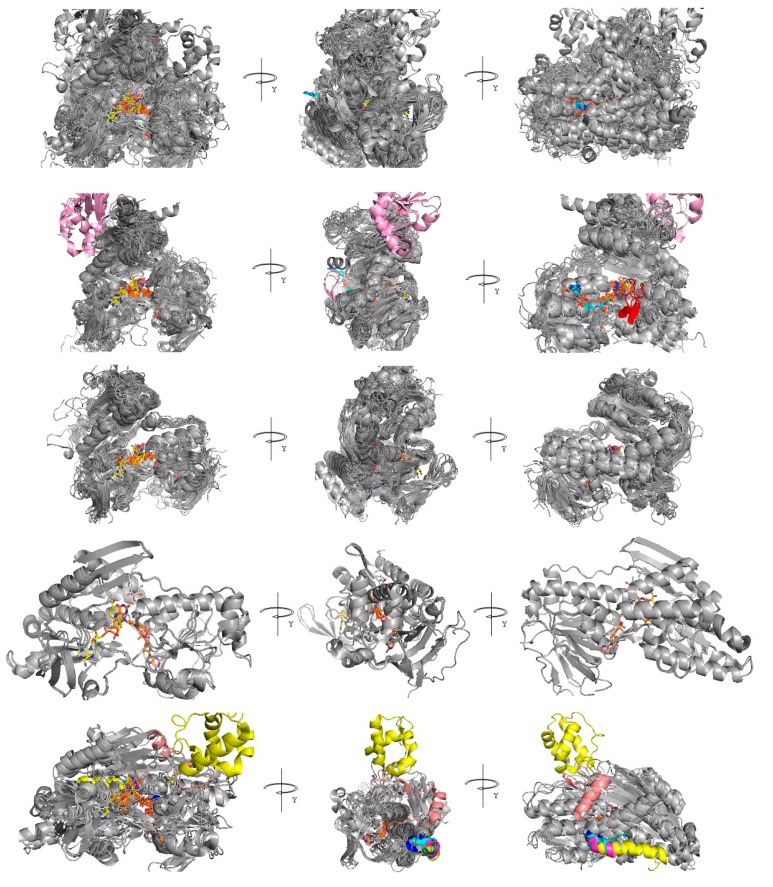
Comparative analysis of AIF/NDI/NDH-2/DLD structures. First row: Superimposition of all the sampled 49 crystallized structures (see Table 1). Second to fifth row: superimposition of all the AIF-like proteins, DLD-like proteins, NDI-like proteins, and NDH-2-like proteins, respectively. All the sampled proteins are reported as grey cartoon representations. Specific protein features are colored according to what is reported in the main text at each row. FAD, NADH, UQ, and CoA are reported as orange, yellow, white, and cyan sticks, respectively, where available at the reported crystallized structures, according to Table 1.

Cartoon representations of the superimposed AIF, NDI, and NDH-2, and all the pGenTHREADER-sampled structures are reported in Figure 4.

Although a common structural trend among all the sampled structures is observed, it is possible to recognize at least three main groups with specific structural features consisting of AIF-like proteins, NDH-2/NDI-like proteins (each with specific features), and lipoamide dehydrogenase-like proteins. The features of each cited group reflect their distribution in the phylogenetic tree (Supplementary Figure S1) built starting from the MSA of the sequences sampled by pGenTHREADER, where it is possible to observe three main groups consisting of AIF-like proteins (including ferredoxin reductases and pyridine nucleotide disulphide CoA NADPH dehydogeanses), NDI-like/NDH-2-like proteins; and other FAD-dependent dehydrogenases (including lipoamide dehydrogenases, thioredoxin reductases, and glutathione reductases).

In detail, the AIF-like protein group contains 20 of the 49 sampled structures. The functional annotation of these 20 sampled structures includes AIF, NADH-oxidase, ferredoxin reductase, rubredoxin reductase, putidaredoxin reductase, and pyridine nucleotide CoA disulfide reductase. The overall structure of those 18 structures overlaps with the human AIF, with an RMSD ranging between 1.06 and 3.1 Å (Table 1). The good superimposition allows to highlight a common cavity hosting a FAD molecule in 14 crystallized structures, a NADH molecule in 2 crystallized structures, and a CoA molecule in 2 crystallized structures, according to Table 1.

A peculiar β-sheet motif located at residues 190–200 of the human AIF (Figure 4) is observed as a specific AIF feature.

Two further structures, namely 3ics.pdb and 3ntd.pdb, annotated as CoA-disulfide reductases, may be associated to the AIF-like protein group. Both the structures host a FAD molecule and a CoA molecule similar to what was observed for 4fx9.pdb and 3cgb.pdb. Nevertheless, both 3ics.pdb and 3ntd.pdb show a 100-aa longer C-terminal domain (residues 461–554 for 3ics.pdb; 471–565 for 3ntd.pdb). Notably, 5vn0.pdb also hosts an oxygen molecule within 3.5 Å from FAD.

The NDH-2/NDI-like protein group contains 7 of the 49 sampled protein structures. Five structures are from bacterial proteins and the other two are from fungal proteins.

The functional annotation of the five sampled bacterial protein structures includes typeII NADH dehydrogenase and sulphide: UQ oxido reductases (Table 1). The related overall structures overlap with the *C. thermarum* NDH-2, with an RMSD ranging between 0.82 and 3.33 Å. The good superimposition allows to highlight a common cavity hosting a FAD molecule in all the five sampled crystallized structures and a NADH molecule in one crystallized structure, according to Table 1.

Notably, 3hyw.pdb hosts a quinone derivative (decylubiquinone, DCQ) and an H_2_S molecule (within 3.5 Å from FAD), whereas 5n1t.pdb (a type II NADH dehydrogenase from *Thioalkalivibrio paradoxus*) was crystallized in complex with CytC and copper chaperone [44]. The main differences between the five structures are at the level of their C-terminus portion. Notably, 5jwc.pdb [45] shows a 70-aa long extra loop (residues 363–433, 5jwc.pdb numbering (Figure 4)).

The two NDI-like proteins are both NADH dehydrogenases crystallized from *S. cerevisiae.* The overall structure of the two proteins (as expected from two crystals of the same protein, crystallized in the presence of similar ligands, i.e., UQ-like molecule and stigmatellin) is very similar, with an estimated RMSD of 0.49 Å. From their superimposition, a common cavity hosting an FAD molecule and a NADH molecule is observed. 4g73.pdb hosts a quinone derivative, whereas 5yjw.pdb [46] hosts a stigmatellin bound at the same level of the quinone-binding region. Notably, stigmatellin is known as a competitive NADH-dehydrogenase inhibitor [47] (Figure 4).

The third group of FAD-dependent dehydrogenases contains 19 dehydrogenase structures, with an overall structure more similar to the ones shown by AIF-like proteins. The functional annotation of these 19 sampled structures includes lipoamide dehydrogenases, glutathione amide reductases, thioredoxin reductases, flavoprotein disulfide reductases, mercuric reductases, and NADPH:2-ketopropyl-coenzyme M oxidoreductase/carboxylases (2-KPCC).

The overall structure of those 19 proteins overlap with the human AIF, with an RMSD ranging between 3.22 and 4.84 Å. The further good superimposition allows to highlight a similarly located common cavity hosting a FAD molecule in 16 crystallized structures and a NADH molecule in 2 crystallized structures, according to Table 1.

Notably, 4m52.pdb hosts a sulfonamide derivative [48] located 9 Å far from the FAD molecule (at a region 3 Å far from the UQ-binding region observed in proteins of the previous groups) whereas 1mo9.pdb hosts a ketopropylthioethanesulphonate [49] bound at the same level of the CoA molecule-binding region observed in the first group of protein structures (Figure 4).

Three further structures, namely 4up3.pdb, 5u63.pdb, and 1ps9.pdb annotated as thioredoxin reductases (the first two) or 2,4-dienoyl-CoA reductase (the latter), may be associated to the AIF-like protein group.

Indeed, all the structures host a FAD molecule and a NAD(P)+ molecule. Nevertheless, 4up3 and 5u63 completely lack a region corresponding to the AIF C-terminal domain, shown by all the AIF-like proteins (residues 491-C-ter, 4bur residues numbering, Supplementary Figure S3), whereas 1ps9 contains a different greater domain in correspondence of the N-terminal domain (residues 1–331, 1ps9 residues numbering). 4up3, 5u63, and 1ps9 were maintained in our comparative analyses as outliers for comparative purposes.

### 3.4. FAD and NADH Binding Regions

**Figure 5:**
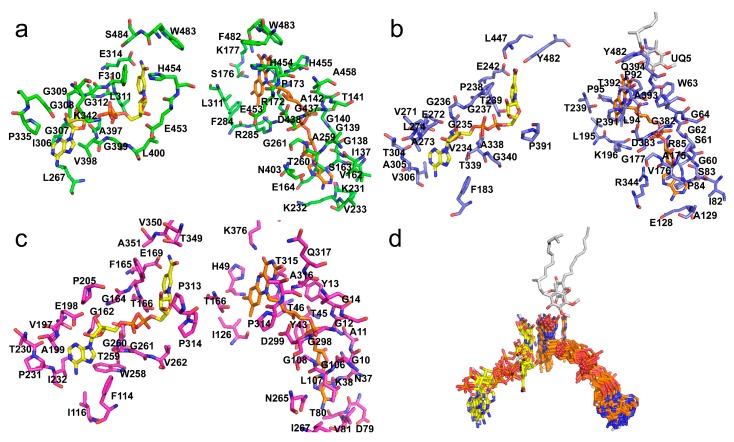
AIF/NDI/NDH-2 cofactor-binding regions. Panels (**a**–**c**) Residues within 4 Å from NADH (yellow sticks, corresponding to NADH_a_ according to NADH cofactor nomenclature reported in [26]) or FAD (orange sticks) for AIF (green sticks from 4bur.pdb), NDI (blue sticks from 4g73.pdb), and NDH-2 (magenta sticks from 5kmr.pdb) are reported and labeled. Panel (**d**) Zoomed-in view of cofactor coordinates from all the investigated proteins obtained by superimposing the sampled crystallized structures (see Table 1). UQ (from 4g73.pdb, corresponding to UQ_I_, according to UQ cofactor nomenclature reported in [25]) and DCQ (from 3hyw.pdb) are reported as white sticks. See Supplementary Figure S7 for visualizing the other cofactors observed along the superimposition of the investigated crystallized FAD/NADH oxidoreductases (i.e., heme C, CoA, O_2_, and H_2_S).

Beyond the highly similar overall structures shared between AIF (4bur.pdb), NDH-2 (5kmr.pdb), and NDI (4g73.pdb), highlighting a similarly located binding region for FAD and NADH, we also observed that the three superimposed crystal structures also bind those cofactors with glycine residues of the fifth sequence motif and a set of conserved aromatic/basic/acidic residues (Figure 4 and Figure 5) located among the fifth and ninth sequence motif.

The variability observed in the composition of FAD- and NADH-binding regions does not alter the ability of those pockets in efficiently binding the two cofactors, as much as most of the sampled FAD/NADH dependent dehydrogenase structures show a FAD (32 out of the 49 compared crystal structures, see Table 1) and a NADH (8 out of the 49 compared crystal structures, see Table 1, in correspondence of NADH_b_, according to NADH cofactor nomenclature reported in [26]) molecule at a very similar position (Figure 5D; Supplementary Table S3).

In general, all the investigated dehydrogenase 3D structures show several conserved residues, aligning with residues of the proposed 10 sequence motifs (Figure 2 and Supplementary Figure S2), interacting with FAD and NADH cofactors (Supplementary Table S3), despite the relatively low overall percentage of identical residues (15%–30% range, Supplementary Table S2, on the full length sequences) shared with AIF/NDI/NDH-2 sequences.

### 3.5. UQ Binding Site Comparative Analyses between AIF and NDH-2

Given the crystallization of an UQ-like molecule both in *S. cerevisiae* NDI (4g73.pdb) and in the sulphide: quinone oxidoreductase from *Aquifex aeolicus* (3hyw.pdb), it is also possible to propose residues putatively involved in the UQ binding in orthologous NDH-2/NDI-like proteins sampled from *Bacteria*/*Fungi* (Table 1).

Notably, for an investigation of the UQ-binding region from *Fungi* NDI-like proteins, it will be sufficient to superpose the crystallized dehydrogenase from *S. cerevisiae* to a comparative model of the fungal protein under investigation and highlight residues within 4 Å from the ubiquinone obtained from the *S. cerevisiae* NDI-crystallized structure (the ones in correspondence of UQ_I_, according to UQ cofactor nomenclature reported in [25]).

Residues forming the UQ-binding region in NDH-2-like crystallized structures were proposed by superimposing the sampled NDH-2-like structures (Table 1) on *A. aeolicus* sulphide: quinone oxidoreductase and by highlighting residues within 4 Å from DCQ crystallized in complex with the structure of sulphide: quinone oxidoreductase from *A. aeolicus* (Supplementary Table S4).

Notably, residues involved in the UQ-binding region in NDI from *Fungi* and NDH-2-like proteins from *Bacteria* are located between the 9th and 10th sequence motifs and the C-terminal portion.

Given the highly similar overall structure observed between AIF, DLD, and NDH-2/NDI-like proteins and based on recent findings about AIF proteins [11,12], it is proposed that UQ-like molecules may participate in the function of AIF-like proteins and DLD-like proteins. Residues proposed to participate in UQ-like molecule-binding regions in AIF-like proteins and DLD-like proteins were highlighted by selecting residues within 4 Å from the two UQ-like molecules entrapped within AIF-like proteins and DLD-like proteins, after superimposition of the two protein sets with 4g73 and 3hyw (Supplementary Table S4).

Notably, residues involved in the potential UQ-binding region in AIF-like proteins and DLD-like proteins from *Bacteria*, *Metazoa*, and *Plants* are located between 8th, 9th, and 10th sequence motifs and the protein C-terminal portions.

### 3.6. Small Molecules and Other Cofactor-Binding Regions

It should be noticed that most of the crystallized structures among the AIF-like proteins, NDI-like proteins, and NDH-2-like proteins host other small molecules and cofactors in dedicated binding regions or are crystallized in complex with other small proteins (Table 1).

For example, NADH oxidases (among AIF-like proteins) from *Lactobacillus brevis* (5vn0.pdb) and *Lactobacillus sanfrancensis* (2cdu.pdb) host an oxygen molecule and an ADP molecule, respectively; 4 disulphide oxidoreductases from *Bacillus anthracis* (3cgb.pdb and 3ics.pdb)*, Pyrococcus horikoshii* (4fx9.pdb), and *Shewanella ioihica* (3ntd.pdb), among AIF-like proteins, host a CoA molecule at a similarly located binding region; the NDH-2-like protein from *T. paradoxus* (5n1t.pdb) was crystallized in complex with CytC and COPC, whereas NDI from *S. cerevisiae* was crystallized in complex with stigmatellin (5yjw); and finally, the NDH-2-like protein from *Plasmodium falciparum* (5jwc) was crystallized in complex with RYL-552 (5n.a.fluoron.a.3n.a.methyln.a.2n.a.{4n.a.(4n.a.(trifluoromethoxy)benzyl)phenyl}quinolinn.a.4(1H)n.a.one).

Notably, also DLD-like proteins were crystallized in complex with small molecules, cofactors, and small proteins, i.e., lipoamide DH from *Mycobacterium tubercolosis* (4m52.pdb) was crystallized in complex with sulphonamide; DLD from *Pseudomonas putida* (6awa.pdb) hosts an AMP molecule; glutathione reductase from *Streptococcus pyogenes* (6n7f.pdb) hosts a riboflavin molecule; NADPH:2-ketopropyl-coenzyme M oxidoreductase from *Xhantobacter autotrophicus* (1mo9.pdb) hosts a ketopropylthioethanesulphonate molecule; whereas DLD from *Geobacillus stearothermophilus* (1ebd.pdb) and thioredoxin reductase from *P. falciparum* (4j56) were crystallized in complex with a fragment of dihydrolipoamide acetyltransferase and thioredoxin, respectively.

While AMP, ADP, riboflavin, and stigmatellin were crystallized in binding regions generally involved in cofactor binding, or overlapping regions, it was observed that disulfide reductases, among AIF-like proteins, host a CoA molecule in a specific binding region consisting of the residues shown in Supplementary Table S5.

Conversely, it was observed that RYL-552 (a quinolinic derivative, see Table 1 and [45]) within NDH-2-like protein from *P. falciparum* (5jwc), SL827 (a sulphonamide derivative, see Table 1 and [48]) within lipoamide DH from *M. tubercolosis* (4m52.pdb), and ketopropylthioethanesulphonate within NADPH:2KPCC oxidoreductase from *Xanthobacterautotrophicus* (1mo9.pdb, [49]) are located at dedicated binding regions involving several species-specific residues not involved in the binding of NADH/FAD/UQ cofactors.

Notably, RYL-552 binds three different binding regions within 5jwc far from the NADH/FAD/UQ cofactor-binding area. While two of those regions appear to be poorly resolved, the third ones appears to be a specific binding cavity [45]. By superimposing 5jwc with the analyzed AIF, NDI, and NDH-2, it appears that NDH-2 and NDI might form several interactions with RYL-552 involving 8 and 11 residues, respectively, that appear to be further well conserved in the sampled NDH-2 and NDI-like proteins (although in the latter, the overlapping binding region appears more buried from the local secondary structure), at variance with the AIF showing a lower number of interacting residues (just four, see Supplementary Table S6 and Supplementary Figure S4). Although AIF-like proteins do not show a well-defined binding pocket in correspondence of RYL-552, at variance with DLD-like proteins showing a sterically hindered/buried region in correspondence withRYL-552, it cannot be excluded that RYL-552 analogs may also bind AIF similarly located accessible binding cavities (Supplementary Figure S4 and Supplementary Figure S5).

The SL827 (a sulphonamide derivative) binding region observed in the lipoamide DH 4m52 consists of few amino acids that appear to be conserved in DLD-like proteins and AIF-like proteins. Notably, by superimposing 4m52 with AIF, NDI, and NDH-2, it appears that NDH-2 and NDI, in correspondence with the sulphonamide derivative binding region, shows a buried region occupied by an a-helical region that most likely will not allow the sulphonamide derivative to penetrate NDI and NDH-2 cofactor-binding regions or affect NDI/NDH-2 activity (Supplementary Figure S4).

Conversely, AIF-like proteins (i.e., 4bur) and DLD-like proteins show an accessible cavity in correspondence with the sulphonamide derivative binding region observed in 4m52 (Supplementary Table S7), making us to purpose that sulphonamide derivatives may target both DLD-like and AIF-like proteins.

The ketopropylthioethanesulphonate (KPC)-binding region observed in 1mo9 (the 2KPCC) consists of few amino acids that appear to be conserved in DLD-like proteins and AIF-like proteins, similar to what observed for sulphonamide. Thus, also, in this case, NDI and NDH-2 show a buried region not accessible to KPC, whereas AIF-like proteins (i.e., 4bur) and DLD-like proteins show an accessible cavity in correspondence with the KPC-binding region observed in 1mo9 (Supplementary Table S7).

Notably, by superimposing DLD-like proteins with disulfide reductases it is possible to observe that the sulphonamide derivative and the KPC bind DLD-like proteins in correspondence of theCoA-binding region detected in disulfide reductases (Supplementary Figure S4).

### 3.7. Small Protein Subunit-Binding Regions

Some of the sampled dehydrogenases were also crystallized in complex with other related protein subunits. i.e., it was observed that flacocytochromecsulphide dehydrogenase from *T. paradoxus* (5n1t.pdb) was crystallized in complex with CytC and a dimeric copper-binding protein (COPC), whereas thioredoxin reductase from *P. falciparum* (4j56.pdb) and DLD from *G.stearothermophilus* (1ebd.pdb), among DLD-like structures, were crystallized in complex with thioredoxin and dihydrolipoamide acetyltransferase, respectively.

**Figure 6:**
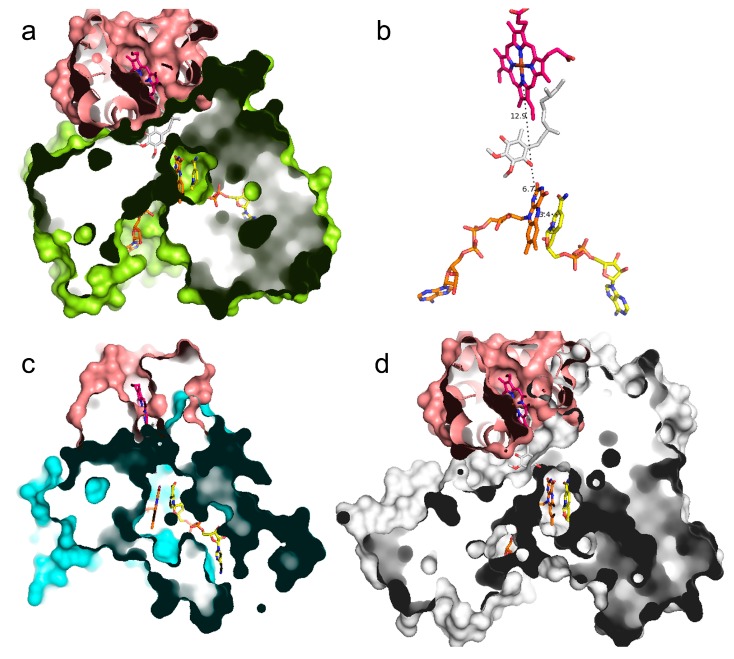
AIF/NDI/NDH-2 possible protein–protein interaction surfaces. Panel (**a**) Zoomed-in views of the crystallized heterodimeric structure of flavoprotein dehydrogenase (yellow surf representation) and CytC (pink surf representation) from *T. paradoxus* (5n1t.pdb) is reported in complex with FAD, (orange sticks), the heme C center in magenta sticks and NADH and UQ (yellow and white sticks, respectively, obtained by superimposition with 4g73 (see methods)). Panel (**b**) FAD, NADH, heme C center (from 5n1t.pdb), and UQ (superimposed from 4g73.pdb) are reported in stick representations. Intermolecular distances are reported by dashed lines and are labeled. Panel (**c**,**d**) 3D models of a putative heterodimeric structure of AIF (4bur.pdb) or NDI (4g73) proteins (cyan or white cartoon, respectively) in complex with CytC from *T. paradoxus* (5n1t.pdb). FAD, NADH, and UQ are reported in stick representations (see previous panels for colors).

By superimposing flavoCytC: sulphide dehydrogenase from *T. paradoxus* with AIF and NDI, it is possible to highlight a binding region of AIF and NDI that is aligned with the flavoCytC: sulphide dehydrogenase protein region involved in interactions with CytC. We observed that the two regions consist of several amino acids located at (or very close to) the 1st, 3rd, 9th, and 10th sequence motifs and at the C-terminal region (Supplementary Table S8 and Figure 6).

From the DLD from *G.stearothermophilus* (1ebd.pdb) crystallized in complex with dihydrolipoamide acetyltransferase, it appears that the 41-aa-long dihydrolipoamide acetyl transferase binding domain may bind at the DLD monomer–monomer interface at the 1ebd C-terminal domain. Some residues close to the 9th and 10th sequence motifs are involved in acetylase binding (Supplementary Table S9)

Similarly, by superimposing one of the sampled thioredoxin reductases to the thioredoxin reductase from *P. falciparum*(4j56.pdb) crystallized in complex with thioredoxin, it is possible to predict a putative binding region for thioredoxin in all the sampled thioredoxin reductases. Notably, by superimposing 4j56 to 4bur, 4g73, and 5kmr, it appears that several residues of 4bur are in the interaction range (below 4 Å) from thioredoxin, whereas 4g73 and 5kmr do not show more than four residues below 4 Å from thioredoxin (Supplementary Table S10).

## 4. Discussion

Among the investigated FAD-dependent dehydrogenases, it is possible to recognize AIF, NADH oxidases/nitrile reductases, ferredoxin reductases, rubredoxin reductases, toluene 2,3-dioxygenase reductases, putidaredoxin reductases, pyridine nucleotide-CoA disulfide oxidoreductases, NDH-2 and NDI, flavocytochrome c sulfide dehydrogenases, sulfide: quinone oxidoreductases, DLD, glutathione amide reductases, thioredoxin reductases, mercuric reductases, and NADPH:2-ketopropyl-CoM oxidoreductase/carboxylases.

Among the 49 sampled dehydrogenase structures, 20 show an AIF-like structure, 5 show an NDH-2-like structure, 2 show an NDI-like structure, and 19 show a DLD-like structure.

By superimposing all the investigated dehydrogenase structures, it is possible to observe that all of them have the same overall structure. Moreover, 46 out of the 49 investigated dehydrogenases host a FAD molecule in the same position, whereas 9 of the 49 host an NADH molecule in the same position.

Notably, 3 out of the 49 dehydrogenase structures host a UQ-like molecule, whereas 4 out of the 49 host a CoA molecule at two dedicated similarly located binding regions.

### 4.1. A Similarly Located FAD/NADH-Binding Region for All the Investigated Flavoprotein Oxidoreductases and New Clues about a Putative UQ-Binding Region

Our analysis has allowed to predict the exact localization of NADH and FAD-binding regions among orthologous sequences of the investigated AIF-like, NDI-like, and NDH-2-like proteins. NADH and FAD-binding regions are also similarly located within DLD-like proteins. Furthermore, based on NDI and NDH-2 available structures, MSA, and recently published functional studies about AIF dehydrogenase activity [11], it may be speculated that AIF proteins may also bind a UQ molecule to participate to oxidative pathways crucial for mitochondrial respiration. Notably, our analysis has allowed to propose the existence of a putative binding region for UQ within the different investigated FAD/NADH-dependent oxidoreductases, including AIF-like proteins, that do not show a solved UQ molecule in the available crystallized structures. Considering DLD, it was already proposed that UQ might be reduced by lipoamide DH [50], although there is no evidence of a putative DLD UQ-binding region. Based on our comparative analyses, it may be speculated that the UQ-binding region within DLD is at the same level of the UQ-binding region highlighted within AIF after superimposition with NDI/NDH-2.

### 4.2. Concerns about the Opportunity to Draw New Inhibitors to be Used as Antibiotic/Antiparasitic Drugs, Directed Against the Investigated FAD/NADH Dehydrogenases

New insights have been acquired about the investigated species-specific FAD/NADH dependent oxidoreductases that raise the question about the opportunity to draw new antibiotic/antiparasitic drugs directed against the cited FAD/NADH-dependent oxidoreductases.

Currently, some of the described FAD/NADH-dependent oxidoreductases are considered in microbiology a crucial target of antibiotics or chemotherapeutics (i.e., those structurally related to RYL-552, a quinolinic derivative or to SL827, a sulphonamide derivative [10,48,51,52,53]) because those enzymes play a fundamental role for the ATP synthesis and oxidative pathways in most microorganisms. The lack of NDH-2-orthologous enzymes in *Mammalia* was retained an important proof of the selective action of potential drug development [10,54,55,56,57].

Although RYL-552 and SL827 appear to target specific binding regions within NDH-2/NDI and DLD bacterial proteins, respectively, given the high structural similarity and the similarly located cofactor-binding regions highlighted in the investigated FAD/NADH-dependent oxidoreductases, it will be necessary to ascertain the absence of interactions with the human AIF/DLD-accessible cavities, before using RYL-552/SL827 structurally related ligands in new preclinical/clinical trials.

Similarly, SL827 and KPC analogs were proposed as chemicals for selective targeting of *M. tuberculosis* DLD and *X. autotrophicus* NADPH-CoM oxidoreductase. However, human DLD/AIF proteins show accessible cavities in correspondence of theSL827/KPC-binding regions observed within *M. tuberculosis* DLD and *X. autotrophicus* NADPH-CoM oxidoreductase. Thus, for SL827/KPC structurally related ligands, it will also be necessary to exclude putative interactions with human AIF/DLD proteins to guarantee the microorganism selectivity of both molecules and to avoid undesirable side effects due to interactions with the human mitochondrial FAD/NADH-dependent oxidoreductases. Notably, antibiotics that might be considered structurally related to SL827, such as sulfamethoxazole/trimethoprim and sulfisoxazole, although commercialized for several years, are known for their serious side effects [58]. It cannot be excluded that those adverse reactions may be ascribed to interactions between the cited antibiotics and AIF/DLD human proteins.

Finally, it should also be noticed that CoA disulfide reductases, structurally related to AIF/DLD-like proteins, host a CoA molecule in correspondence of the SL827/KPC-binding region. The presence of a CoA molecule in CoA disulfide redutases, at the level of the SL827/KPC-binding region observed in the homologous AIF/DLD-like proteins, raises the question about possible competitive effects between SL827/KPC structurally related ligands and disulfide reductases CoA-binding regions that may decrease the efficiency and selectivity of SL827/KPC structurally related ligands.

### 4.3. New Clues in Support of AIF Participation in Mitochondrial Respiration

Based on the performed comparative analyses, it was possible to propose that flavoprotein oxidoreductase interaction surfaces are involved in the binding of other protein subunits involved in respiratory mechanisms (i.e., a putative AIF-binding region for CytC, after comparison with flavocytochrome c sulfide DH from *T. paradoxus*).

Considering (a) the role played by NDH-2/NDI-like proteins in bacterial/fungal respiration [7,25], (b) the direct interaction between a *T. paradoxus* flavoprotein oxidoreductase and a CytC-like protein [44], and (c) the recently proposed rotenone sensitive NADH:UQ oxidoreductase activity of AIF [11,12], it could be speculated that a direct interaction between AIF and CytC and other mitochondrial copper-binding proteins of the inner membrane [59,60,61] might also exist in *Metazoan* mitochondria, with several implications regarding AIF participation in mitochondrial respiration. These data are coherent with previous observations about alternative respiratory protein complexes [62,63,64,65].

Concerning the above reported hypothesis, we should recall that the incomplete NADH consumption along mitochondrial respiration assays performed in the presence of high concentrations of rotenone and other respiratory protein–complex inhibitors, as well as the protective role played by AIF in the maintenance of respiratory chain complexes, are well documented [22,66,67,68].

Thus, it appears that other mitochondrial enzymes may contribute to the buffering of altered NAD^+^/NADH ratios by oxidizing alternatively cytosolic NADH or mitochondrial matrix NADH, and here, we might speculate that AIF, being anchored to the mitochondrial inner membrane, protruding into the intermembrane space, may participate in the regulation of the NAD^+^/NADH or FAD/FADH2 (maybe UQ/UQH2) ratio. AIF participation in the regulation of redox signaling between mitochondria and other cell compartments may be crucial above all in those tissues in which the G3P shuttle [69], nicotinamide nucleotide transhydrogenase (NNT) [70], NADH-b5 oxidoreductase [71,72], or malate/aspartate shuttle [15,73,74] do not work properly or when the cited proteins, mitochondrial oxidative phosphorylation complex subunits, or matrix proteins are mutated or downregulated [75,76]. If AIF behaves as a NADH dehydrogenase, able to oxidize cytosolic NADH, it may also participate in reprogramming metabolic pathways, providing new clues towards the full comprehension of the puzzling Warburg effect condition [77].

Notably, quinoline 3-sulfonamides were recently proposed to be a class of high-affinity (bioactive in the nM range) lactic dehydrogenase inhibitors [78] that may be employed in cancer therapies. However, quinoline 3-sulfonamides are ligands structurally related both to RYL-552 and, although at a lower extent, to quinone-related structures (i.e., stigmatellin or menaquinone) known to be able to inhibit or bind NDI/NDH-2-like proteins. Thus, it appears that it will be necessary in the near future to ascertain putative activity/affinity of quinoline 3-sulfonamides for NDI/NDH-2 and also for AIF/DLD-like proteins.

**Figure 7:**
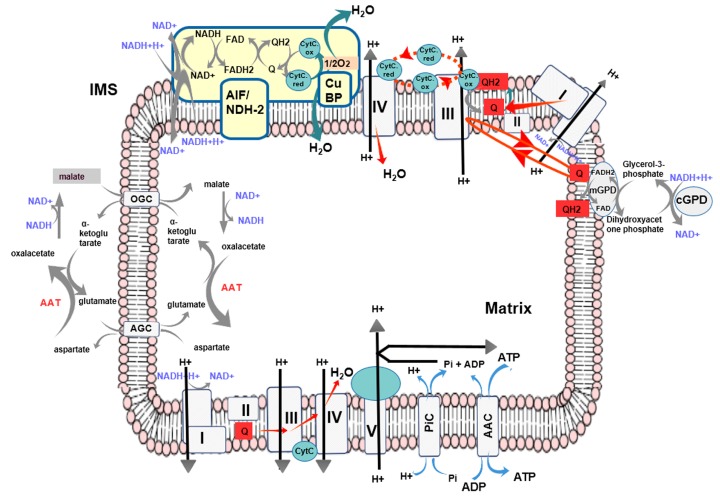
Scheme representation describing the putative participation of AIF to mitochondrial respiration. Cofactors involved in the reaction NAD^+^ to NADH, FAD to FADH2, a quinone derivative (Q) to the corresponding quinol derivative (QH2), and CytC.ox to CytC.red are indicated by labels. Respiratory chain complexes (I, II, III, and IV) and the ATP-synthase, ADP/ATP Carrier (AAC), phosphate carrier (PiC), malate/aspartate shuttle protein members (with the two carriers OGC and AGC) and the glycerol-3-phosphate dehydrogenase (GPD) shuttle members are reported to show a putative context of action in mitochondrial redox pathways for AIF. A putative copper-binding protein (CuBP) is also indicated. IMS = Intermembrane Space.

Based on the above reported observations, the redox path proposed in Figure 7, and the available crystallized structures, we propose that NDH-2, NDI, and AIF proteins may have two binding regions for NADH and two further binding regions for UQ-analogous molecules to work correctly, according to what proposed by [25,26]. The need for having two molecules for each cofactor may reflect the necessity to bind to specific binding pockets with different affinities for the entry/exit of oxidized/reduced cofactors, similar to what proposed for other quinol-dependent oxidases [30,79].

Notably, Ferreira et al. [26] proposed that a high NADH/NAD^+^ ratio may facilitate the formation of the AIF: NADH complex, which may trigger AIF dimerization and cell death induction, through the cleavage of the first 102 residues of AIF [22,26], the following release of AIF_∆1-102_ from mitochondria, and its translocation to the nucleus, where AIF (complexed with MIF [24]) mediates chromatinolysis. This hypothesis was based on AIF-crystallized structures solved by Ferreira et al. [26]. In detail, Ferreira et al. have provided a crystal structure of AIF in the presence of one FAD molecule (that they called the oxidized hAIF_∆1-102ox_, available as a single monomer under the PDB code 4bv6.pdb, [26]), and one further AIF structure in the presence of both FAD and NADH (that they called the reduced hAIF_∆1-102rd_: NAD(H), available as a dimer of homodimers under the PDB code 4bur.pdb, [26]).

Both the obtained crystallized structures 4bur and 4bv6 lack a protein region at the level of the protein segment including residues 509–560, defined by the authors as the apoptogenic segment [26]. In detail, 4bur lacks the 517–552 region (see 4bur, chain B), whereas 4bv6 lacks atomic coordinates for the 546–558 protein region (see the single chain of 4bv6). It should be noticed that the missing regions are close to the NADH_b_-binding region (according to NADH cofactor nomenclature reported in [26])and in correspondence with theUQ_II_NDH-2-binding region (according to UQ cofactor nomenclature reported in [25]).

In light of these observations and according to [26], it might also be speculated that NADH presence (i.e., a high NADH/NAD^+^ ratio) can make more exposed/accessible AIF redox catalytic sites for UQ-like ligands that may participate in the re-oxidation of NADH mediated by FAD. Conversely, a high NAD^+^/NADH ratio may induce conformational changes that lower the affinity of NADH and UQ-like molecules for AIF catalytic pockets.

Nevertheless, Miseviciene et al. [12] showed that AIF has quinone reductase activity (expressed as the *k_cat_/K_m_* ratio) 10^2^- to 10^4^-fold lower than that of cytochrome P450 reductase and NAD(P)H: quinone oxidoreductase (NQO1) and 10^1^- to 10^2^-fold lower than that of *Mammalian* thioredoxin reductase but is comparable with the activity of glutathione reductase from various sources (see [12] and references therein).

However, the low quinone reductase activity exerted by the recombinant AIF investigated by Miseviciene et al. may also in part be ascribed to the fact that Miseviciene et al. used for their analyses the AIF_∆1-77_ protein domain [12] instead of the AIF_∆1-52_ protein domain, be the latter the complete protein domain responsible for redox activity [22,80]. It will be necessary to use AIF_∆1-52_ recombinant protein for checking/re-estimating AIF quinone reductase activity and compare it with the recently quinone reductase activity mediated by the *Mammalian* complex I [81].

Concerning the dimerization process, it should also be noticed that, beyond missing residues at the 509–560 AIF region, both 4bur and 4bv6 [12] crystallized structures lack the first 124 and thus it cannot be excluded that the missing fragments at the C-terminal domain (at the level of residues 509–560) and at the N-terminal domain may facilitate a non-physiological multimerization process.

In support of the lack of clarity about dimerization processes and cofactor access, it is observed that in correspondence of the protein region containing the missing portions in 4bur/4bv6 (residues 509–560), 4bv6 also shows the crystallized C-terminal region (Ile610, 4bv6 residues numbering) located between the two termini (Pro545 and Asp559, 4bv6 residues numbering) of the non-solved portion. Furthermore, the mercuric reductase (4k7z.pdb) from *P. aeruginosa* hosts a mercuric ion in a region partially overlapping with the AIF 546-558 (4bv6 residues numbering) protein region.

All these observations make possible to hypothesize the putative involvement of a metal ion that might locate at the level of the AIF 546-558 (4bv6 residues numbering) missing residues. The presence of a metal ion working as a further cofactor might reveal crucial for the correct protein function and/or redox mechanism.

More in general solving the structure of the missing fragments at the N-/C-terminal regions and at the level of 509-560 AIF protein region will help in elucidating conformational changes responsible both for cofactor entry in the AIF catalytic site and dimerization/multimerization processes.

On this concern it should finally be noticed that the proposed distance between FAD and UQ (obtained by superimposition as previously described) as well as the distance between UQ and CytC (obtained by superimposition as previously described) in AIF would be below 13 Å, which is in the useful distance-range for allowing electron tunneling [30], although the proposed AIF-UQ-binding region appears to be less accessible than its counterpart in NDH-2 and NDI. Also, the presence of an oxygen molecule (obtained by superimposition with 5vn0.pdb, as previously described) as well as an H_2_S molecule (3hyw.pdb) in the investigated crystallized structures (see Supplementary Figure S7) makes us think that several investigated flavoprotein dehydrogenases may show in the future other unsuspected abilities.

### 4.4. Pieces of Evidence about the Possible Targeting of AIF for the Development of New Treatments for Mitochondrial Dysfunction in Rare Diseases

If AIF participation to mitochondrial respiration would be ascertained, AIF protein may be considered as a target for the stimulation of mitochondrial function for the development of new treatments for diseases characterized by mitochondrial dysfunction.

If an UQ structurally related ligand-binding region would be highlighted experimentally on the recombinant protein, according to what reported above, we would expect that several UQ structurally related drugs used for the treatment of mitochondrial diseases may improve patient conditions by stimulating mitochondrial function through AIF. On this concern, it could be speculated that UQ structurally related approved drugs, like EPI-743, Idebenone [82], and KH176 [83], may exert their effect also by targeting the AIF UQ-derivative-binding region.

At the moment, it is proposed that EPI-743 and Idebenone may act on complex I [47,84] whereas KH-176 would interact with the thioredoxin system [83].

Nevertheless, if complex I subunits are mutated, above all, if mutations are located close to the proposed CoQ-binding region, it would be difficult that a greater availability of the cited cofactors (or structurally related ligands) might rescue complex I defect, acting directly on the same complex I activity.

Conversely, because of the similarity between KH-176 (and its precursors, i.e., Trolox), EPI-743, Idebenone, and UQ, it may be speculated that KH-176, EPI-743, and Idebenone may target an UQ analog-binding region in a functional protein participating to mitochondrial respiration.

According to [83], the most probable binding region of KH-176 could be located within the thioredoxin reductase (TrxR1). Nevertheless, TrxR1 shows a highly similar overall structure with AIF-like proteins and appears to be even more similar to DLD-like proteins (Supplementary Figure S6). Thus, it might be speculated that KH-176 may also target AIF and DLD, beyond TrxR1, at the proposed similarly located UQ-binding region (Supplementary Table S4).

### 4.5. Conclusions

The presented computational strategy, based on the combination of sequence database screening and fold recognition methods, will allow clinicians and biomedical researchers to identify/highlight “structurally related proteins”, shared from *Bacteria*, *Protista*, *Fungi*, *Metazoan*, and *Plants*, not detectable using only sequence alignment-based search tools, to choose the target of new drugs under investigation with greater confidence aiming to reduce off-target effects.

The possibility to target a species/specific enzyme becomes crucial in the battle against antibiotic resistance. Indeed, it is known that several *Bacteria* have become resistant to antibiotics directed against enzymes involved in cell wall synthesis, cell membrane function, protein and nucleic acid biosynthesis, and antimetabolites.

To overcome the problems related to antimicrobial resistance, medical scientists are starting to target the cellular energy-generating machinery [78] and, in general, enzymes regulating crucial respiration/oxidative pathways in pathogenic microorganisms [54,85,86,87].

Notably, new chemicals directed against FAD/NADH oxidoreductases are already approved drugs and/or are being tested in preclinical/clinical trials [53,82,83]. Given our findings, clinicians and biomedical researchers could now test drugs designed for the targeting of FAD/NADH oxidoreductases (NDH-2/TrxR1 like, from *Bacteria*, *Fungi*, or *Protista*) on human mitochondria to exclude deleterious interactions with human mitochondrial proteins (i.e., AIF, DLD, and TrxR1), ascribable to the common origin shared by mitochondria from *Metazoans* and *Protista* [88] and, in general by mitochondria and *Bacteria* [89,90].

At the same time, the proposed strategy will allow clinicians and biomedical researchers to link putative adverse effects, following the administration of new drugs directed against bacterial/fungal/protista FAD/NADH oxidoreductase (NDH-2/TrxR1-like) proteins to unpredicted interactions with human structurally related AIF/DLD/TrxR1.

Furthermore, the proposed comparative analyses might help clinicians and medical researchers to evaluate the possibility of considering FAD/NADH oxidoreductases as a possible target of new small molecules that are able to modulate mitochondrial respiration in patients affected by rare diseases or cancers characterized by a severe mitochondrial dysfunction.

## 5. Patents

The presented data are part of the Italian patent: Pierri et al., 2019; Computational methods for the identification of FAD/NADH dehydrogenases binding regions for drug design and discovery; IT Patent 102019000022545.

## Figures and Tables

**Table 1:** List of the investigated homologous crystallized structures and specific structural features. The proteins sampled using pGenTHREADER together with the RMSD between coordinates of the sampled structure backbones superimposed to the structure of the three query sequences (AIF (4bur.pdb); NDI (4g73.pdb); and NDH-2 (5kmr.pdb)) are reported. The best hit of each reported structure sampled by Blastp in *H.sapiens*, *C. thermarum*, and *S. cerevisiae* is also reported. For each best hit E-value, %ID and query coverage are also reported. Abbreviations: AIF, apoptosis-inducing factor; DH, dehydrogenase; LD, lipoamide dehydrogenase; DLD, dihydrolipoyl dehydrogenase or dihydrolipoamide dehydrogenase; OX, oxidase; RED, reductase; GLR1, glutathione disulfide reductase; Trx, Thioredoxin; mt, mitochondrial; TRR1, Trx-disulfide reductase; RYL-552, 5n.a.fluoron.a.3n.a.methyln.a.2n.a.{4n.a.(4n.a.(trifluoromethoxy)benzyl)phenyl}quinolinn.a.4(1H)n.a.one); SL827, N~2~-((2-amino-5-bromopyridin-3-yl)sulfonyl)-N-(4-methoxyphenyl)-N~2~-methylglycinamide; KPC, ketopropylthioethanesulphonate; CytC, Cytochrome C.

	Functional Annotation	Res. N.	Organism	Crystallized cofactors	Crystallized Inhibitors	AIF (4bur)	NDH2-(5kmr)	NDI (4g73)	*H. sapiens* Blast Hits					*C. thermarum* Blast Best Hit					*S. cerevisiae* S288C Blast Best Hit				
						RMSD (Å)			Protein Name	Accession	Query cover	E-val	%ID	Protein Name	Accession	Query cover	E-val	%ID	Protein Name	Accession	Query cover	E-val	%ID
**AIF-like structures**																							
**4bur**	AIF	511	*H.sapines*	FAD/NADH	n.a.	0	3.12	2.22	mt isoform AIF-exB	NP_665811.1	100%	0.0	100%	n.a.	n.a.	n.a.	n.a.	n.a.	Irc15p	NP_015308	25%	0.98	25.55%
**5fs8**	AIF	474	*H.sapines*	FAD	n.a.	1.06	2.71	1.82	mt isoform AIF	NP_004199.1	99%	0	99.61%	n.a.	n.a.	n.a.	n.a.	n.a.	n.a.	n.a.	n.a.	n.a.	n.a.
**5vn0**	NADH OX	449	*L.brevis*	FAD/NADH/O_2_	n.a.	2.28	2.03	2.18	protein transport protein Sec23A	NP_006355.2	100%	0.0	100%	CoA-disulfide reductase	WP_007505374.1	2%	3 × 10^−86^	50.00%	GTPase-activating protein SEC23	NP_015507.1	98%	0.0	49.80%
**1xhc**	NADH OX /nitrile RED	555	*P. furiosus*	FAD	n.a.	1.799	2.443	2.458	AIF 3 isoform 2	NP_001018070.1	73%	9 × 10^−20^	28.47%	CoA-disulfide RED	WP_007505374.1	98%	9 × 10^−92^	35.83%	Aif1p	NP_014472.1	54%	9 × 10^−11^	26.36%
**2bc0**	NADH OX	473	*S. pyrogens*	FAD	n.a.	1.53	1.859	1.658	AIF 3 isoform 1	NP_653305.1	67%	2 × 10^−18^	25.75%	CoA-disulfide RED	WP_007505374.1	92%	2 × 10^−74^	31.29%	GLR1	NP_015234.1	40%	8 × 10^−10^	27.09%
**2cdu**	NAD(P)H OX	452	*L. sanfrancensis*	FAD/ADP	n.a.	2.226	2.2	2.095	mt isoform AIF	NP_004199.1	56%	2 × 10^−11^	25.46%	CoA-disulfide RED	WP_007505374.1	96%	3 × 10^−74^	31.52%	GLR1	NP_015234.1	39%	3 × 10^−8^	27.66%
**1nhs**	NADH PerOX	447	*E. fecalis*	FAD	n.a.	1.738	1.932	2.041	AIF 3 isoform 1	NP_653305.1	55%	7 × 10^−22^	27.17%	CoA-disulfide RED	WP_007505374.1	98%	9 × 10^−92^	35.83%	GLR1	NP_015234.1	39%	8 × 10^−4^	22.40%
**3lxd**	Ferredoxin RED	409	*E. coli*	n.a	n.a.	1.949	2.842	2.606	AIF 3 isoform 1	NP_653305.1	89%	8 × 10^−51^	30.24%	CoA-disulfide RED	WP_007505374.1	80%	6 × 10^−26^	29.48%	GLR1	NP_015234.1	35%	2 × 10^−3^	24.03%
**3fg2**	Ferredoxin RED	404	*R. palustris*	FAD	n.a.	1.633	2.087	2.340	AIF 3 isoform 1	NP_653305.1	91%	6 × 10^−47^	29.22%	CoA-disulfide RED	WP_007505374.1	77%	7 × 10^−22^	24.15%	GLR1	NP_015234.1	32%	4 × 10^−3^	25.90%
**2gqw**	Ferredoxin RED	401	*Pseudomonas sp. KKS102*	FAD	n.a.	1.986	2.362	2.365	AIF 3 isoform 2	NP_001018070.1	91%	5 × 10^−27^	28.39%	CoA-disulfide RED	WP_007505374.1	52%	3 × 10^−14^	29.78%	Irc15p	NP_015308.1	24%	6 × 10^−3^	28.85%
**2v3a**	Rubredoxin RED	381	*P. aeruginosa*	FAD	n.a.	2.278	3.067	6.698	AIF 2	NP_001185625.1	61%	3 × 10^−3^	24%	CoA-disulfide RED	WP_007505374.1	71%	2 × 10^−23^	27.02%	n.a.	n.a.	n.a.	n.a.	n.a.
**3klj**	NADH:rubredoxinoxidoRED	378	*C. acetobutylicum*	FAD	n.a.	2.498	2.745	2.335	AIF 3 isoform 2	NP_001018070.1	95%	5 × 10^−27^	23.41%	CoA-disulfide RED	WP_007505374.1	89%	1 × 10^−21^	23.69%	mRNA-binding ubiquitin-specific protease UBP3	NP_011078.3	7%	0.77	53.57%
**3ef6**	Toluene 2,3-Dioxygenase RED	400	*P. putida*	FAD	n.a.	1.914	1.979	2.146	AIF 3 isoform 1	NP_653305.1	83%	7 × 10^−44^	33.14%	CoA-disulfide RED	WP_007505374.1	43%	9 × 10^−15^	33.33%	Aif1p	NP_014472.1	48%	6 × 10^−4^	25.35%
**1q1r**	Putidaredoxin RED	421	*P. putida*	FAD	n.a.	1.581	2.85	3.069	AIF 3 isoform 3	NP_001139760.1	87%	5 × 10^−40^	28.95%	CoA-disulfide RED	WP_007505374.1	72%	9 × 10^−25^	27.74%	GLR1	NP_015234.1	38%	2 × 10^−4^	21.64%
**3oc4**	Pyridine nucleotide-disulfide oxidoRED	422	*E. faecalis*	FAD	n.a.	3.143	2.451	3.207	AIF 3 isoform 1	NP_653305.1	62%	4 × 10^−13^	25.26%	CoA-disulfide RED	WP_007505374.1	95%	4 × 10^−45^	26.53%	GLR1	NP_015234.1	51%	1 × 10^−5^	23.36%
**3iwa**	Pyridine nucleotide-disulphideoxidoRED	397	*D. vulgaris*	n.a.	n.a.	2.243	3.06	2.664	AIF 3 isoform 2	NP_001018070.1	68%	8 × 10^−24^	28.66%	CoA-disulfide RED	WP_007505374.1	95%	5 × 10^−82^	33.55%	GLR1	NP_015234.1	59%	3 × 10^−9^	22.49%
**3cgb**	Pyridine Nucleotide Coenzyme A-Disulfide RED	444	*B.anthracis*	FAD/CoA	n.a.	2.17	2.37	2.341	glycerol-3-phosphate DH, mt	NP_000399.3	10%	0.8	39.58%	CoA-disulfide RED	WP_007505374.1	91%	5 × 10^−137^	47.05%	Irc15p	NP_015308.1	60%	1 × 10^−5^	23.51%
**4fx9**	CoA disulfide RED	453	*P. horikoshii*	FAD/CoA	n.a.	2.148	2.223	2.488	AIF 3 isoform 1 [Homo sapiens]	NP_653305.1	70%	1 × 10^−22^	27.55%	CoA-disulfide RED	WP_007505374.1	96%	3 × 10^−105^	39.28%	GLR1	NP_015234.1	64%	2 × 10^−12^	24.36%
**3ics**	CoA disulfide RED	555	*B. anthracis*	ADP/FAD/CoA	n.a.	1.845	1.958	1.98	DLD. mt isoform 4	NP_001276681.1	51%	2 × 10^−9^	26.46%	CoA-disulfide RED	WP_007505374.1	75%	3 × 10^−83^	32.13%	thiosulfate sulfurtransferase RDL2	NP_014929.3	10%	1 × 10^−3^	29.73%
**3ntd**	NADH-dependent persulfide RED	565	*S. ioihica*	FAD/CoA	n.a.	1.92	1.95	1.95	AIF mt isoform AIF-exB	NP_665811.1	47%	6 × 10^−10^	28.01%	CoA-disulfide RED	WP_007505374.1	82%	1 × 10^−85^	31.57%	DLD	NP_116635.1	45%	2 × 10^−7^	25.87%
**Type II NADH DH-like structures**																							
**5kmr**	Type II NADH DH	405	*C. thermarum*	FAD/NAD	n.a.	3.12	0	1.31	AIF 2	NP_001185625.1	72%	2 × 10^−10^	25.34%	NAD(P)/FAD-dependent oxidoRED	WP_007502350.1	98%	0.0	100.00%	NADH-ubiquinone RED (H(+)-translocating) NDE1	NP_013865.1	80%	7 × 10^−29^	28.29%
**5n1t**	FlavoCytC sulfide DH	393	*T. paradoxus*	CytC, COPC, FAD	n.a.	2.82	3.33	2.96	n.a.	n.a.	n.a.	n.a.	n.a.	NAD(P)/FAD-dependent oxidoRED	WP_007505419.1	74%	5 × 10^−22^	26.33%	n.a.	n.a.	n.a.	n.a.	n.a.
**5na1**	NADH:quinoneoxidoRED	398	*S. aureus*	FAD	n.a.	2.63	0.82	1.39	n.a.	n.a.	n.a.	n.a.	n.a.	NAD(P)/FAD-dependent oxidoRED	WP_007502350.1	97%	3 × 10^−130^	46.48%	nucleoside triphosphate pyrophosphohydrolase HAM1	NP_012603.1	12%	0.59	34.69%
**5jwc**	Type II NADH DH	495	*P. falciparum*	FAD	RYL-552	3.88	1.68	0.85	AIF 2	NP_001185625.1	6%	1.7	44.44%	NAD(P)/FAD-dependent oxidoRED	WP_042685058.1	49%	1 × 10^−11^	22.68%	NADH-UQ RED (H(+)-translocating) NDE1	NP_013865.1	94%	8 × 10^−60^	30.32%
**3hyw**	Sulfide:quinoneoxidoRED	429	*aeolicus*	FAD/DCQ/H_2_S	n.a.	3.52	2.75	2.86	sulfide:quinoneoxidoRED. mt (Homo sapiens)	NP_001258142.1	68%	3 × 10^−12^	23.70%	NAD(P)/FAD-dependent oxidoRED	WP_007505419.1	75%	4 × 10^−15^	22.82%	NADH-UQ RED (H(+)-translocating) NDE2	NP_010198.1	55%	1 × 10^−9^	27.27%
**Ndi1 - NADH DH like structures**																							
**4g73**	Ndi1 - NADH DH	502	*S. cerevisiae*	FAD/NAD/UQ5	n.a.	2.22	1.31	0	n.a.	n.a.	n.a.	n.a.	n.a.	NAD(P)/FAD-dependent oxidoRED	WP_007502350.1	86%	1 × 10^−30^	25.93%	NADH-UQ RED (H(+)-translocating) NDI1	NP_013586.1	97%	0.0	99.80%
**5yjw**	Ndi1 - NADH DH.	454	*S. cerevisiae*	FAD	Stigmatellin	2.12	1.32	0.49	n.a.	n.a.	n.a.	n.a.	n.a.	NAD(P)/FAD-dependent oxidoRED	WP_007502350.1	90%	7 × 10^−31^	25.93%	NADH-UQ RED (H(+)-translocating) NDI1	NP_013586.1	100%	0.0	100.00%
**Other DH**																							
**4m52**	LD.	465	*M. tubercolosis*	FAD	SL827	4.28	4.341	1.974	DLD. mt isoform 1	NP_000099.2	98%	8 × 10^−87^	35.82%	DLD	WP_007503768.1	96%	2 × 10^−120^	44.81%	DLD	NP_116635.1	96%	3 × 10^−86^	37.63%
**6aon**	DLD	473	*B. pertussis*	n.a.	n.a.	4.84	2.03	3.76	DLD. mt isoform 1	NP_000099.2	98%	7 × 10^−141^	46.74%	DLD	WP_007503768.1	98%	1 × 10^−106^	40.46%	DLD	NP_116635.1	97%	3 × 10^−143^	47.81%
**4jq9**	DLD	471	*E. coli*	FAD	n.a.	3.61	2.168	2.238	DLD. mt isoform 1	NP_000099.2	94%	2 × 10^−113^	43.61%	DLD	WP_007505013.1	95%	3 × 10^−121^	43.74%	DLD	NP_116635.1	93%	3 × 10^−100^	40.79%
**6awa**	DLD	475	*P. putida*	FAD/AMP	n.a.	3.47	2.6	2.67	DLD. mt isoform 1	NP_000099.2	96%	1 × 10^−153^	50.43%	DLD	WP_007505013.1	97%	6 × 10^−121^	43.19%	DLD	NP_116635.1	97%	1 × 10^−140^	46.47%
**5j5z**	DLD	477	*H. sapiens*	FAD	n.a.	3.23	2.77	3.13	DLD. mt isoform 1	NP_000099.2	95%	0.0	99.97%	DLD	WP_007505013.1	92%	2 × 10^−108^	42.30%	DLD	NP_116635.1	93%	0.0	57.17%
**1zmd**	DLD	474	*H. sapiens*	FAD/NAD	n..a.	3.27	2.83	2.77	DLD. mt isoform 1 (Homo sapiens)	NP_000099.2	100%	0.0	99.79%	DLD	WP_007505013.1	96%	2 × 10^−110^	42.52%	DLD	NP_116635.1	98%	0.0	57.59%
**5u25**	DLD	478	*N. gonorrhoeae*	FAD	n.a.	3.68	2.44	2.47	DLD. mt isoform 1	NP_000099.2	75%	7 × 10^−98^	39.48%	DLD	WP_007505013.1	77%	2 × 10^−112^	41.76%	DLD	NP_116635.1	76%	2 × 10^−96^	40.17%
**3urh**	DLD	491	*R. meliloti*	FAD	n.a.	3.376	2.29	1.732	DLD. mt isoform 1	NP_000099.2	95%	1 × 10^−179^	55.08%	DLD	WP_007505013.1	94%	3 × 10^−117^	42.58%	DLD	NP_116635.1	94%	2 × 10^−163^	51.37%
**1lvl**	LD	458	*P. putida*	FAD/NADH	n.a.	3.664	2.976	4.089	DLD. mt isoform 1	NP_000099.2	98%	5 × 10^−90^	38.09%	DLD	WP_007505013.1	99%	2 × 10^−132^	43.94%	DLD	NP_116635.1	98%	1 × 10^−81^	36.86%
**1ebd**	DLD	455	*G. stearothermophilus*	FAD/Dihydrolipoamide acetyltransferase	n.a.	3.22	2.19	3.69	DLD.mt isoform 1	NP_000099.2	98%	3 × 10^−119^	43.89%	DLD	WP_007505013.1	99%	0.0	68.65%	DLD	NP_116635.1	98%	3 × 10^−110^	43.29%
**2yqu**	LD	455	*T. thermophilus*	FAD	n.a.	3.884	3.548	2.97	DLD. mt isoform 1	NP_000099.2	98%	4 × 10^−140^	46.41%	DLD	WP_007505013.1	99%	1 × 10^−115^	43.41%	DLD	NP_116635.1	99%	2 × 10^−143^	48.00%
**3lad**	Lipoamide deydrogenase	476	*A. vinelandii*	FAD	n.a.	4.442	2.221	2.995	DLD. mt isoform 1	NP_000099.2	96%	3 × 10^−150^	49.36%	DLD	WP_007505013.1	98%	3 × 10^−121^	42.55%	DLD	NP_116635.1	98%	3 × 10^−135^	44.61%
**2r9z**	Glutathione amide RED	463	*C. gracile*	FAD	n.a.	2.813	2.396	2.046	glutathione RED. mt isoform 1	NP_000628.2	95%	9 × 10^−147^	48.80%	DLD	WP_007505013.1	94%	2 × 10^−64^	30.25%	GLR1	NP_015234.1	96%	1 × 10^−141^	46.12%
**6n7f**	Glutathione RED	451	*S. pyogenes*	Riboflavin/FAD	n.a.	3.7	2.74	1.98	glutathione RED. mt isoform 1	NP_000628.2	99%	9 × 10^−160^	52.48%	NAD(P)/FAD-dependent oxidoRED	WP_042684715.1	88%	1 × 10^−53^	29.70%	GLR1	NP_015234.1	98%	9 × 10^−149^	48.70%
**5vdn**	Glutathione RED	449	*Y. pestis*	FAD	n.a.	3.73	2.41	2.66	glutathione RED. mt isoform 1	NP_000628.2	96%	1 × 10^−161^	53.90%	DLD	WP_007505013.1	95%	5 × 10^−61^	32.09%	GLR1	NP_015234.1	96%	9 × 10^−149^	50.75%
**4j56**	Trx RED	504	*P. falciparum*	FAD/Trx	n.a.	3.45	3.33	2.01	Trx RED 2. mt isoform 2	NP_001339229.1	91%	6 × 10^−146^	45.13%	DLD	WP_007505013.1	89%	3 × 10^−31^	26.23%	GLR1	NP_015234.1	89%	3 × 10^−74^	33.47%
**1xdi**	Flavoprotein Disulfide RED	499	*M. tuberculosis*	FAD	n.a.	4.29	2.975	3.65	DLD. mt isoform 1	NP_000099.2	91%	2 × 10^−35^	25.49%	DLD	WP_007503768.1	91%	2 × 10^−47^	29.12%	DLD	NP_116635.1	92%	8 × 10^−35^	26.10%
**1mo9**	NADPH:2-ketopropyl-coenzyme M oxidoRED/carboxylase (2-KPCC)	523	*X. autotrophicus*	FAD	KPC	4.724	2.596	3.091	DLD. mt isoform 1	NP_000099.2	87%	4 × 10^−24^	22.88%	NAD(P)/FAD-dependent oxidoRED	WP_042684715.1	69%	2 × 10^−30^	28.65%	DLD	NP_116635.1	86%	2 × 10^−22^	22.13%
**4k7z**	Mercuric RED	467	*P. aeruginosa*	FAD/NADP	n.a.	3.807	3.341	3.498	DLD. mt isoform 1	NP_000099.2	97%	2 × 10^−54^	29.32%	DLD	WP_007505013.1	94%	3 × 10^−71^	35.68%	DLD	NP_116635.1	95%	3× 10^−51^	29.64%
**Outliers**																							
**4up3**	Trx RED	312	*E. histolytica*	FAD/NADPH	n.a.	3.59	3.76	3.76	(F-actin)-monooxygenase MICAL2 isoform f	NP_001269597.1	13%	0.2	30.23%	Trx-disulfide RED	WP_007502507.1	99%	8 × 10^−68^	37.50%	TRR1	NP_010640.1	98%	4 × 10^−138^	60.83%
**5u63**	Trx RED	315	*H. influenzae*	FAD/NADP	n.a.	2.01	3.06	4.393	DLD. mt isoform 1	NP_000099.2	9%	2.2	48.28%	Trx-disulfide RED	WP_007502507.1	97%	3 × 10^−76^	39.43%	TRR1	NP_011974.1	99%	7 × 10^−101^	49.85%
**1ps9**	2,4-dienoyl-CoA RED	671	*E. coli*	FMN/FAD/NADP	n.a.	2.02	3.93	2.16	L-amino-acid OX isoform 2	NP_001244946.1	6%	3.1	40.48%	NADPH DH NamA	WP_007504681.1	49%	1 × 10^−30^	30.00%	NADPH DH	NP_012049.1	34%	3 × 10^−19^	26.98%

## Supplementary Materials

The following are available online at https://www.mdpi.com/2077-0383/8/12/2117/s1, Figure S1: Phylogenetic tree of the crystallized AIF/NDH-2/NDI-homologous proteins, Figure S2: AIF/NDI/NDH-2 conserved sequence motifs within other homologous FAD/NADH-dependent crystallized dehydrogenases, Figure S3: 3D structure of FAD/NADH-dependent dehydrogenases used as outliers, Figure S4: Cartoon representation of the superimposition of AIF, DLD, NDH-2, NADPH-CoMoxidoreductase, CoA disulfide reductase, and NDI in the presence of cofactors NADH, FAD, UQ, and CoA and inhibitors RYL-552, SL827, and KPC, Figure S5: Surf representation of the superimposition of AIF, DLD, NDH-2, and NDI and putative interactions with the quinoline derivative RYL-552, Figure S6: Comparative analysis of AIF/DLD/TrxR1-crystallized structures, Figure S7: Zoomed-in view of FAD/NADH/UQ/HemeC/CoA/O_2_/H_2_S ligand coordinates from all the investigated proteins obtained by superimposing the sampled crystallized structures (see Table 1), Table S1: Similarity/identity matrix of the listed 41 protein sequences sampled by Blastp and used for generating Figure 2 about sequence motifs, Table S2: Similarity/identity matrix of the listed 49 protein sequences sampled by pGenTHREADER, Table S3: FAD and NADH-binding residues of the crystallized structures reported in Table 1 after their superimposition, Table S4:UQ/DCQ-binding residues of the crystallized structures reported in Table 1 after their superimposition, Table S5: CoA-binding residues of the crystallized CoA disulfide reductases reported in Table 1, Table S6: RYL-552-binding residues of the crystallized NDH-2 (5jwc) from *P. falciparum*, Table S7: SL827 and KPC-binding residues within the crystallized DLD (4m52) from *M.tuberculosis* and NADPH-CoM oxidoreductase (1mo9) from *X.autotrophicus*, Table S8:CytC and CopC (copper-binding proteins)-binding residues of the crystallized NDH-2 dehydrogenase (5n1t) from *T. paradox*, Table S9: Lipoamide acetylase-binding residues of the crystallized DLD (1ebd) from *G.stearothermophilus*, Table S10: Thioredoxin reductase-binding residues of the crystallized Trx red (4j56) from *P. falciparum*.

## Abbreviations

AIF	Apoptosis-inducing factor	DH	dehydrogenase
LD	lipoamide dehydrogenase	DLD	dihydrolipoyl dehydrogenase or dihydrolipoamide dehydrogenase
OX	oxidase	RED	reductase
GLR1	glutathione disulfide reductase	Trx	Thioredoxin
mt	mitochondrial	TRR1	Trx-disulfide reductase 1
MSA	multiple sequence alignment	RMSD	root-mean-square deviation
MIF	migration inhibitory factor	RYL-552	5n.a.fluoron.a.3n.a.methyln.a.2n.a.{4n.a.(4n.a.(trifluoromethoxy)benzyl)phenyl}quinolinn.a.4(1H)n.a.one)
SL827	N~2~-((2-amino-5-bromopyridin-3-yl)sulfonyl)-N-(4-methoxyphenyl)-N~2~-methylglycinamide	KPC	Ketopropylthioethanesulphonate
CytC	cytochrome c	NAD^+^	nicotinamide adenine dinucleotide
FAD	flavin adenine dinucleotide	UQ	ubiquinone
CoA	Coenzyme A		
