# Supplementary material for: FAD/NADH Dependent Oxidoreductases: From Different Amino Acid Sequences to Similar Protein Shapes for Playing an Ancient Function
Source: J Clin Med. 2019 Dec 2;8(12):2117. doi: 10.3390/jcm8122117 (PMC6947548; doi:10.3390/jcm8122117)
Supplement: Supplementary file 1 [file jcm-08-02117-s001.zip › Supp.Figures.1-6.pdf]

Supp. Fig. 1

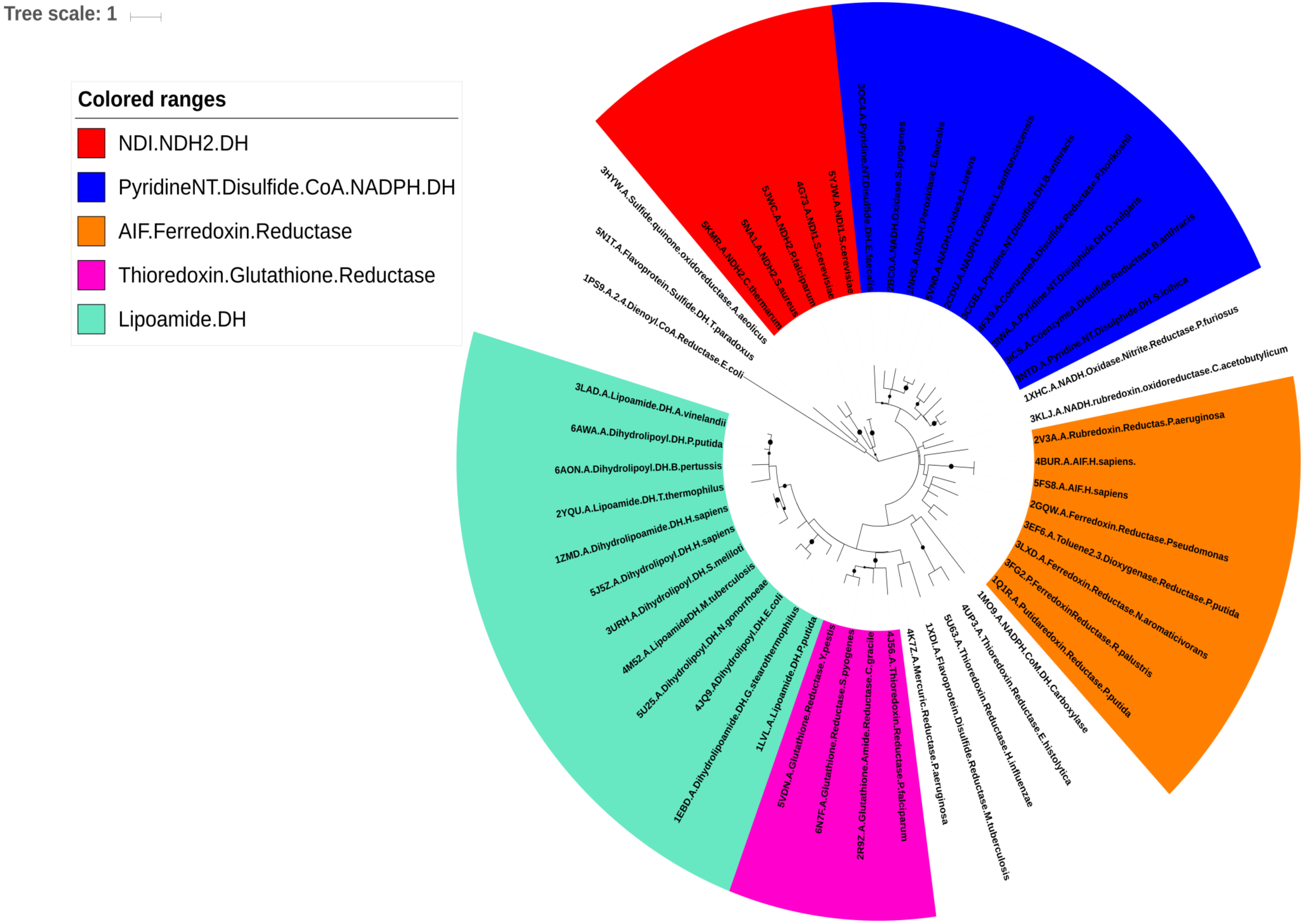

Tree scale: 1
Colored ranges
NDI.NDH2.DH
PyridineNT.Disulfide.CoA.NADPH.DH
AIF.Ferredoxin.Reductase
Thioredoxin.Glutathione.Reductase
Lipoamide.DH
5YJW.A.NDI1.S.cerevisiae
4G73.A.NDI1.S.cerevisiae
5JWC.A.NDH2.P.falciparum
5NA1.A.NDH2.S.aureus
5KMR.A.NDH2.C.thermarum
3HYW.A.Sulfide.quinone.oxidoreductase.A.aeolicus
5N1T.A.Flavoprotein.Sulfide.DH.T.paradoxus
1PS9.A.2.4.Dienoyl.CoA.Reductase.E.coli
3OC4.A.Pyridine.NT.Disulfide.DH.E.faecalis
2BC0.A.NADH.Oxidase.S.pyogenes
1NHS.A.NADH.Peroxidase.E.faecalis
5VN0.A.NADH.Oxidase.L.brevis
3CDU.A.NADPH.Oxidase.L.sanfranciscensis
3CGB.A.Pyridine.NT.Disulfide.DH.B.anthracis
4FX9.A.CoenzymeA.Disulfide.Reductase.P.horikoshii
3IWA.A.Pyridine.NT.Disulphide.DH.D.vulgaris
3ICS.A.CoenzymeA.Disulfide.Reductase.B.anthracis
5NTD.A.Pyridine.NT.Disulphide.DH.S.ioihica
1XHC.A.NADH.Oxidase.Nitrite.Reductase.P.furiosus
3KLJ.A.NADH.rubredoxin.oxidoreductase.C.acetobutylicum
2V3A.A.Rubredoxin.Reductas.P.aeruginosa
4BUR.A.AIF.H.sapiens.
5FS8.A.AIF.H.sapiens
2GQW.A.Ferredoxin.Reductase.Pseudomonas
3EF6.A.Toluene2.3.Dioxygenase.Reductase.P.putida
3LXD.A.Ferredoxin.Reductase.N.aromaticivorans
3FG2.P.FerredoxinReductase.R.palustris
1Q1R.A.Putidaredoxin.Reductase.P.putida
1MO9.A.NADPH.CoM.DH.Carboxylase
4UP3.A.Thioredoxin.Reductase.E.histolytica
5U63.A.Thioredoxin.Reductase.H.influenzae
1XDI.A.Flavoprotein.Disulfide.Reductase.M.tuberculosis
4K7Z.A.Mercuric.Reductase.P.aeruginosa
4J56.A.Thioredoxin.Reductase.P.falciparum
2R9Z.A.Glutathione.Amide.Reductase.C.gracile
6N7F.A.Glutathione.Reductase.S.pyogenes
5VDN.A.Glutathione.Reductase.Y.pestis
1LVL.A.Lipoamide.DH.P.putida
1EBD.A.Dihydrolipoamide.DH.G.stearothermophilus
4JQ9.ADihydrolipoyl.DH.E.coli
5U25.A.Dihydrolipoyl.DH.N.gonorrhoeae
4M52.A.LipoamideDH.M.tuberculosis
3URH.A.Dihydrolipoyl.DH.S.meliloti
5J5Z.A.Dihydrolipoyl.DH.H.sapiens
1ZMD.A.Dihydrolipoamide.DH.H.sapiens
2YQU.A.Lipoamide.DH.T.thermophilus
6AON.A.Dihydrolipoyl.DH.B.pertussis
6AWA.A.Dihydrolipoyl.DH.P.putida
3LAD.A.Lipoamide.DH.A.vinelandii

Supp. Fig. 2

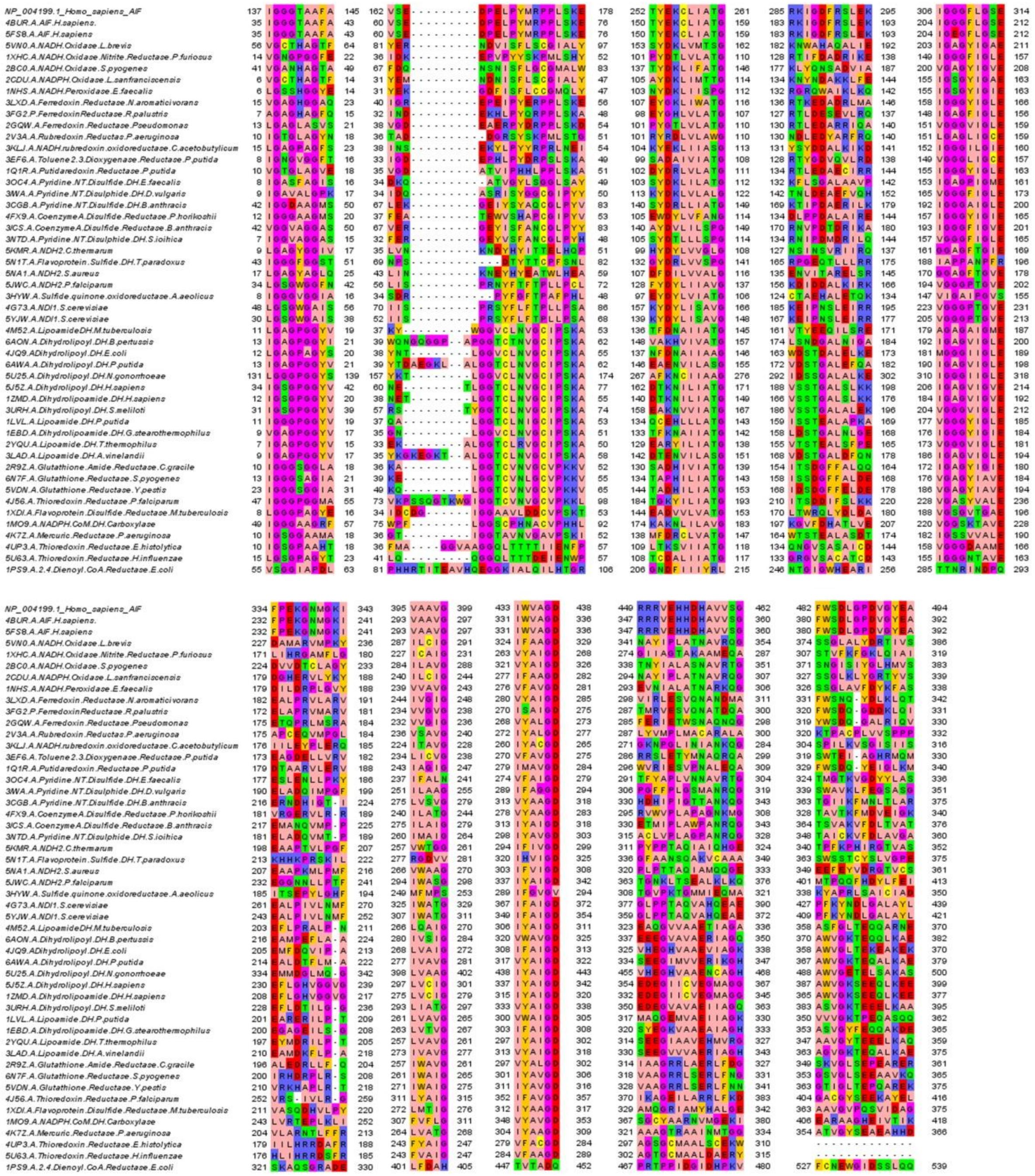

Supp. Fig. 3

a

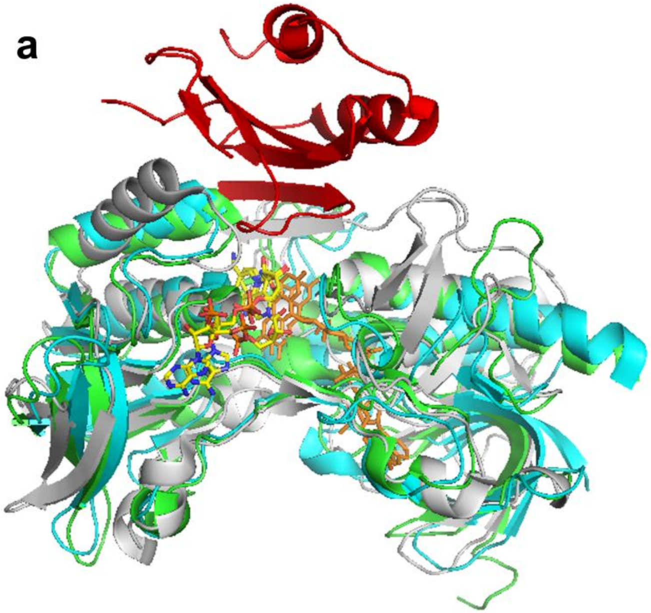

b

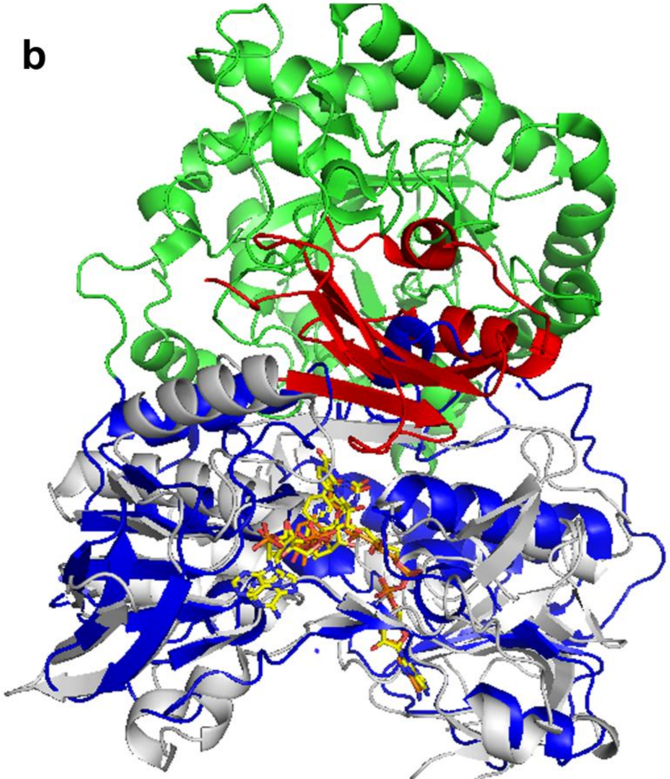

Supp. Fig. 4

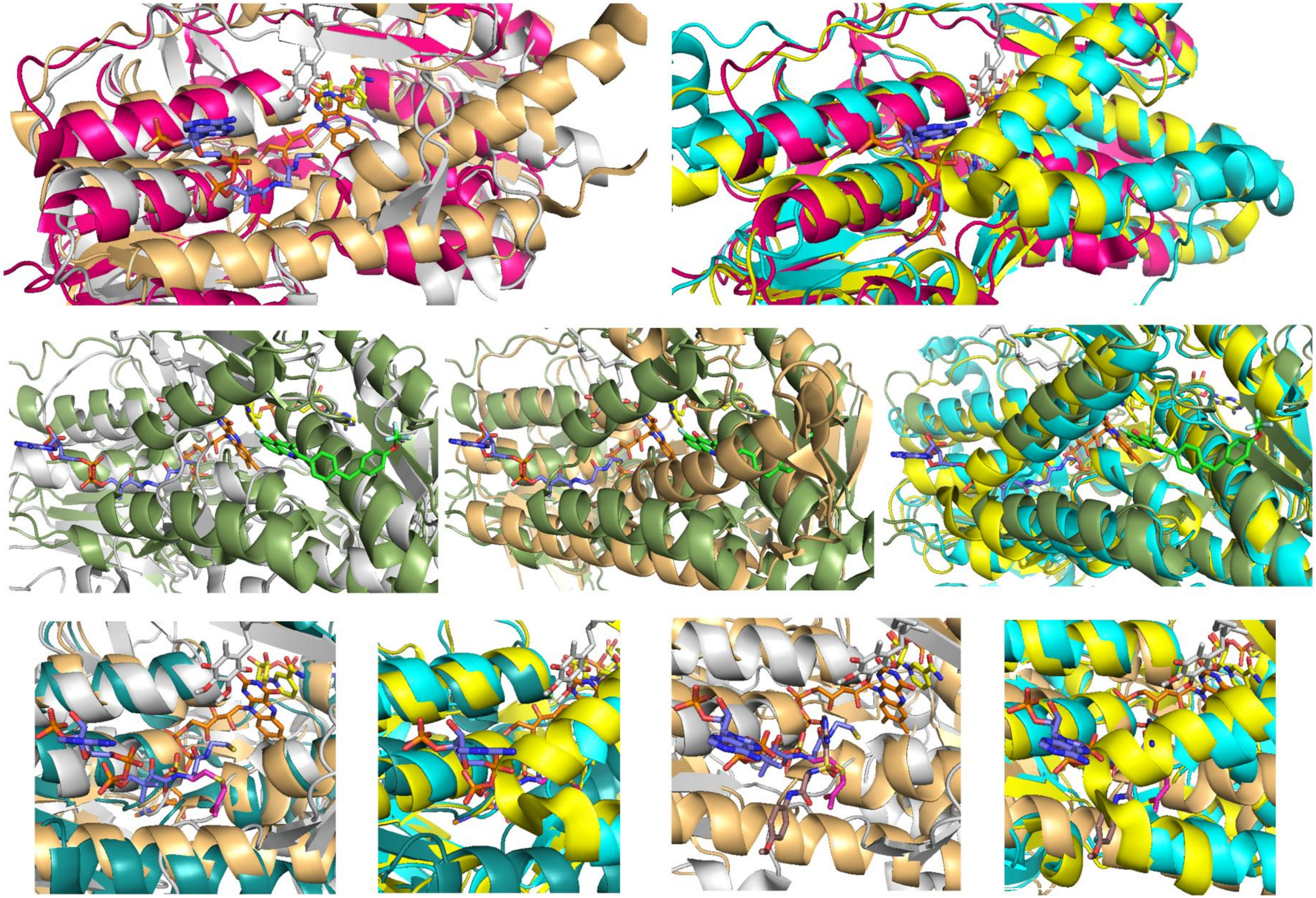

Supp. Fig. 5

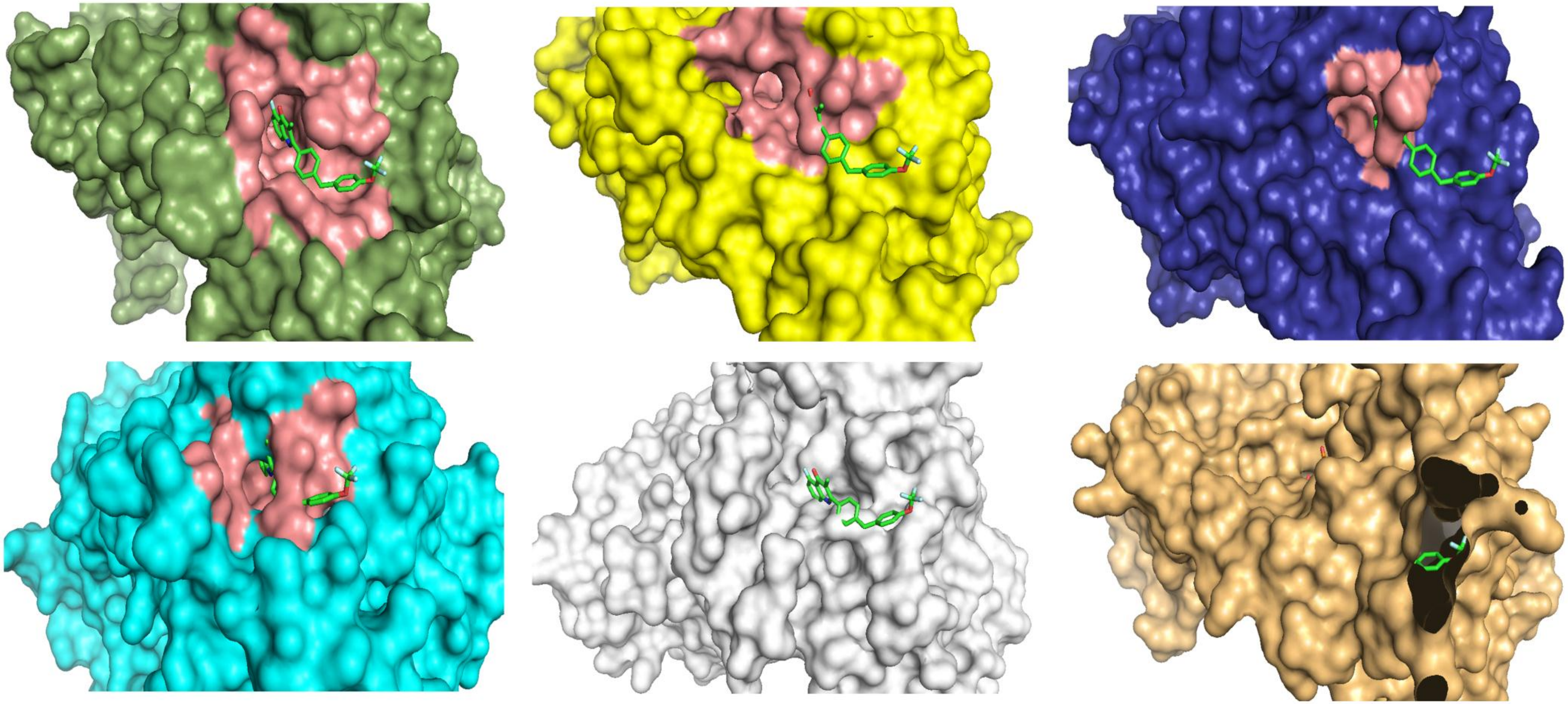

Supp. Fig. 6

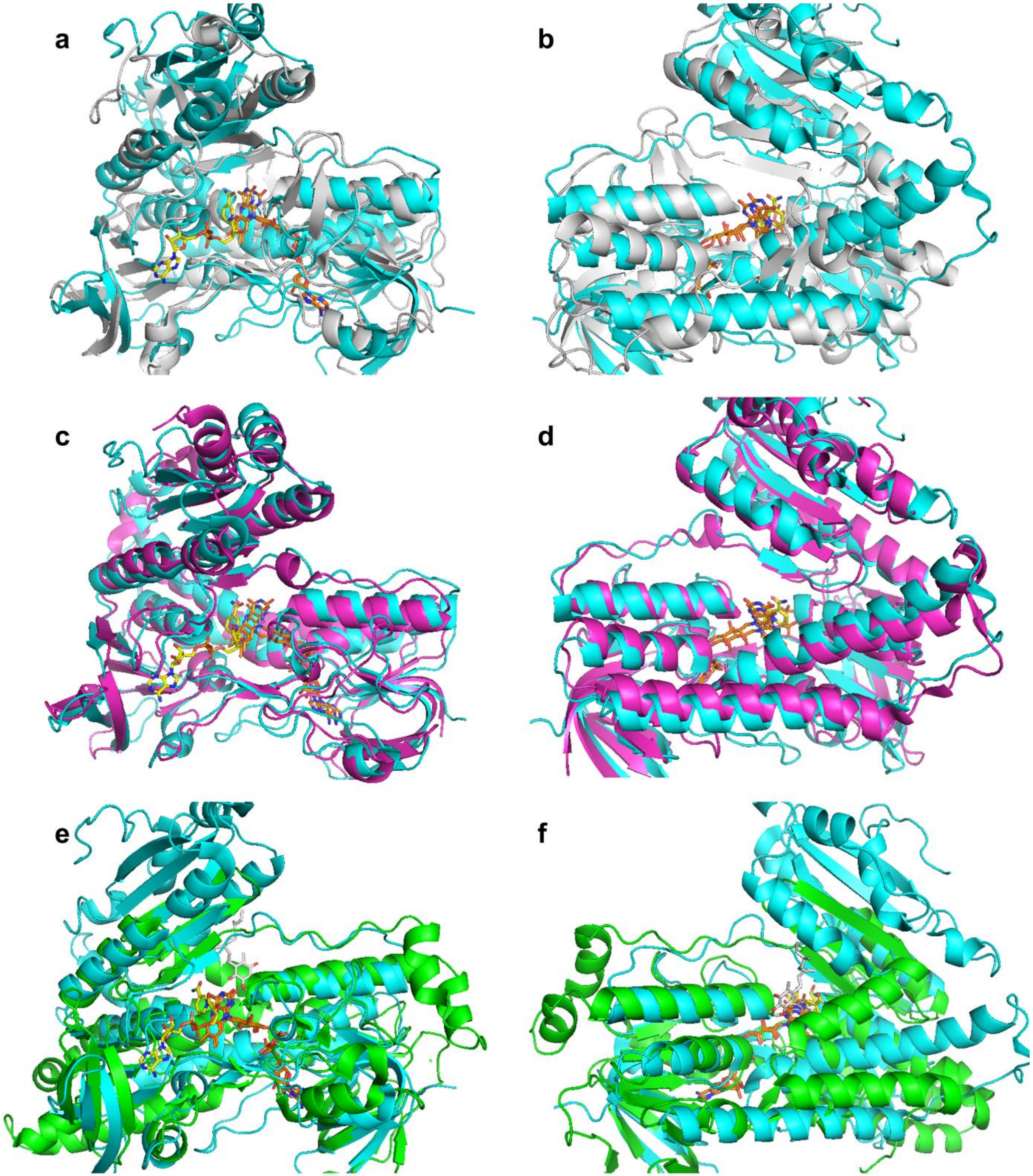

Alternative Fig. 6

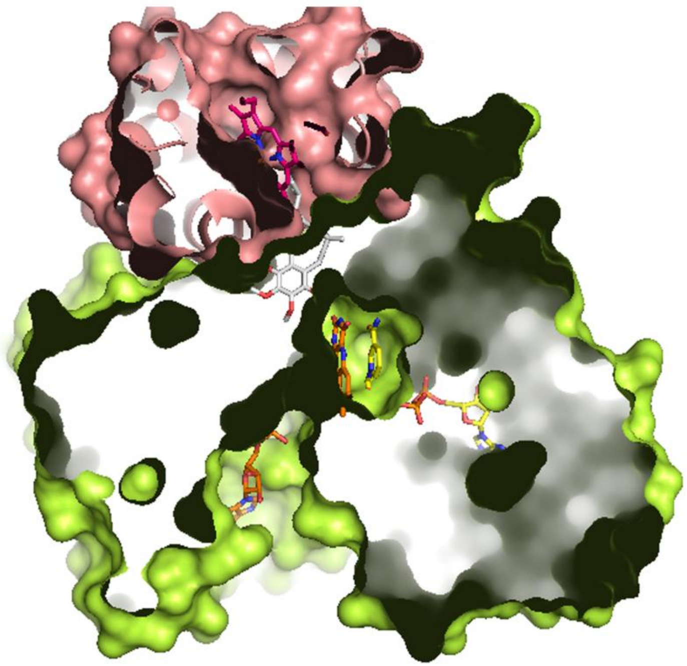

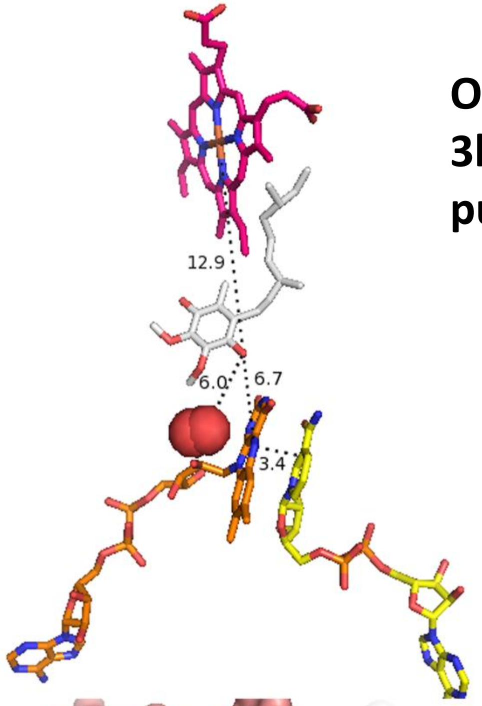

Oxygen from 3hyw.pdb to be put here?

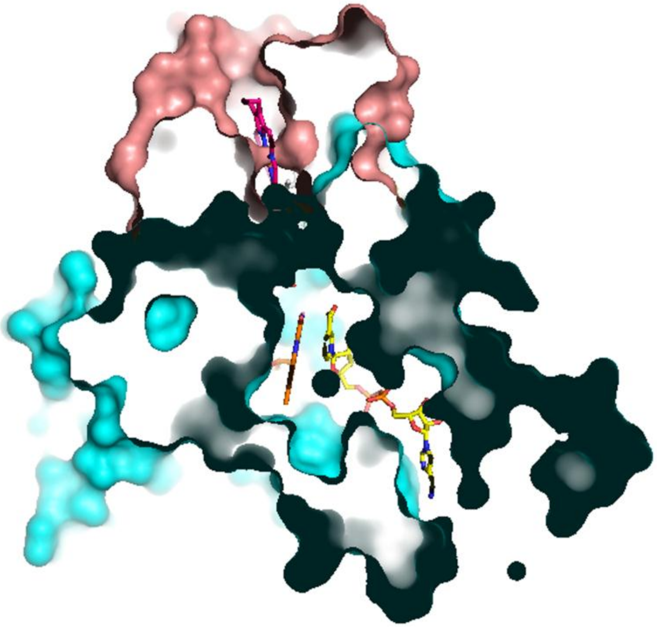

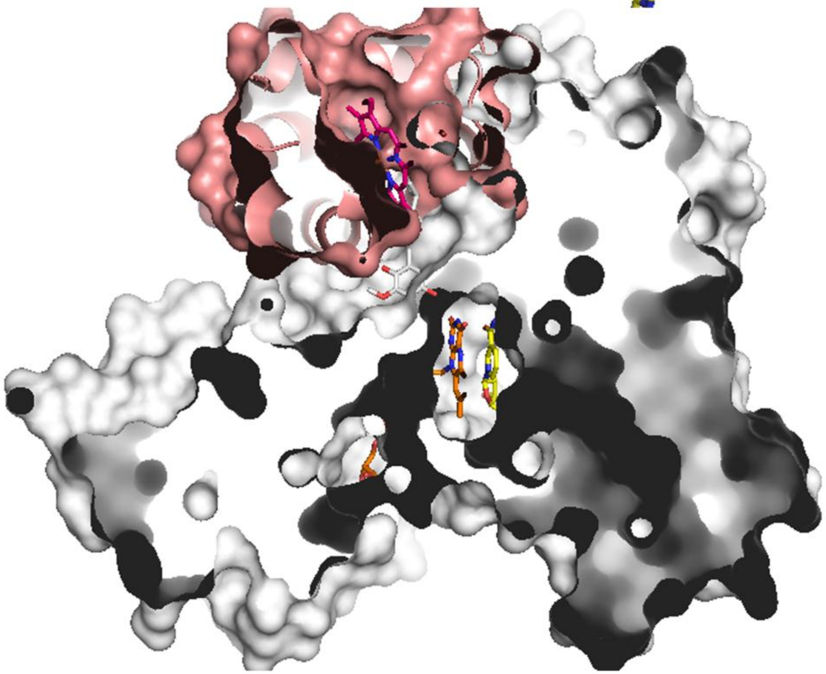

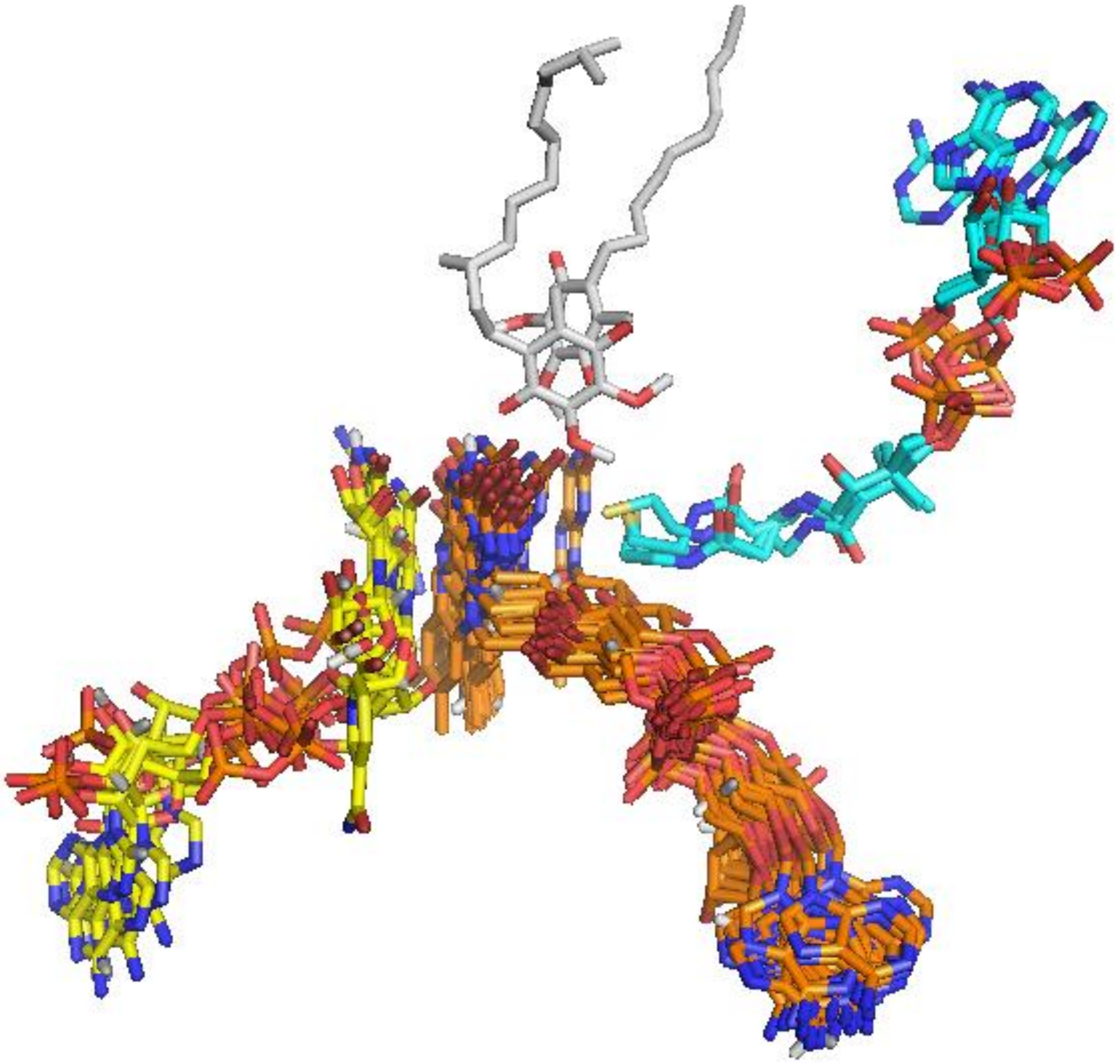

Alternative Panel D for Fig. 5; O2 (5vn0.pdb) or H2S (3hyw.pdb) here?
