# Supplementary figures and images for: FAD/NADH Dependent Oxidoreductases: From Different Amino Acid Sequences to Similar Protein Shapes for Playing an Ancient Function
Source: J Clin Med. 2019 Dec 2;8(12):2117. doi: 10.3390/jcm8122117 (PMC6947548; doi:10.3390/jcm8122117)

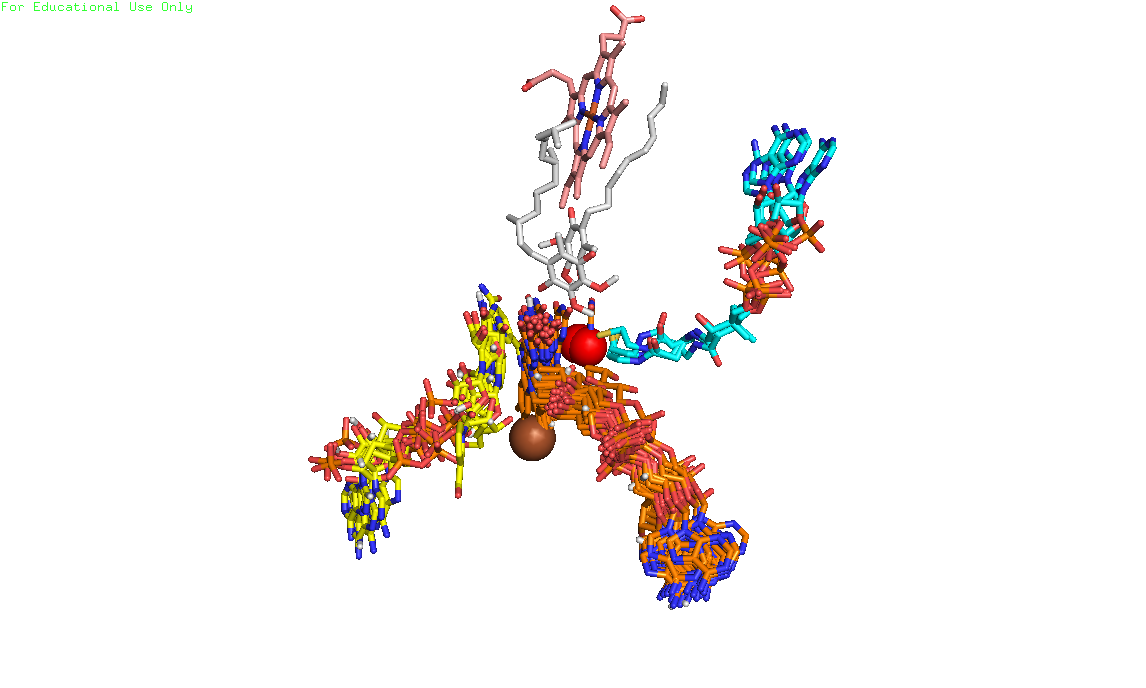

Supplement: Supplementary file 1 [file jcm-08-02117-s001.zip › Supp.Fig.7.png]
